# Why More Leaflets? The Role of Natural Selection in Shaping the Spatial Pattern of Leaf-Shape Variation in *Oxytropis diversifolia* (Fabaceae) and Two Close Relatives

**DOI:** 10.3389/fpls.2021.681962

**Published:** 2021-08-19

**Authors:** Hui Wang, Pei-Liang Liu, Jian Li, Han Yang, Qin Li, Zhao-Yang Chang

**Affiliations:** ^1^College of Life Sciences, Northwest A&F University, Yangling, China; ^2^Key Laboratory of Resource Biology and Biotechnology in Western China, Northwest University, Xi’an, China; ^3^Department of Science and Education, Field Museum, Chicago, IL, United States

**Keywords:** leaf shape, cline, natural selection, gene flow, *Oxytropis*

## Abstract

Leaf shape exhibits tremendous diversity in angiosperms. It has long been argued that leaf shape can affect major physiological and ecological properties of plants and thus is likely to be adaptive, but the evolutionary evidence is still scarce. *Oxytropis diversifolia* (Fabaceae) is polymorphic for leaf shape (1 leaflet, 1–3 leaflets, and 3 leaflets) and exhibits clinal variation in steppes of Nei Mongol, China. With two close relatives predominantly fixed for one phenotype as comparison (*Oxytropis neimonggolica* with 1 leaflet and *Oxytropis leptophylla* with 5–13 leaflets), we used a comprehensive cline-fitting approach to assess the role of natural selection in shaping the spatial pattern of leaf-shape variation in this system. For 551 individuals sampled from 22 populations, we quantified leaf-morphological differentiation, evaluated patterns of neutral genetic variation using five chloroplast DNA intergenic regions and 11 nuclear microsatellite loci, and performed microhabitat and macroclimatic-association analyses. We found that 1-leaflet proportions in *O. diversifolia* populations significantly increased from west to east, and three phenotypes also differed in leaflet-blade size. However, compared with the other two species, populations of *O. diversifolia* showed little neutral genetic differentiation, and no population structure was detected at either marker. We further revealed that the leaf-shape cline could largely be explained by three macroclimatic variables, with leaflet number decreasing and leaflet-blade size increasing with annual precipitation and showing the reverse trends with temperature seasonality and isothermality. Our results suggest that spatially varying abiotic environmental factors contribute to shape the leaf-shape cline in *O. diversifolia*, while the interspecific pattern may be due to both local adaptation and historical events.

## Introduction

Leaf shape is one of the most variable plant traits and exhibits tremendous diversity in angiosperms. Understanding the evolutionary drivers of diversification in leaf shape and their functional significance has been of continuing interest for many years (Nicotra et al., 2011). Remarkable differences in leaf shape may occur between closely related species (e.g., *Pelargonium*, Jones et al., 2009; *Achillea*, Sha et al., 2018), as intraspecific geographic variants (*Asclepias tuberosa*, Wyatt and Antonovics, 1981; *Ipomoea hederacea*, Bright and Rausher, 2008), or even in the same plant (heterophylly and heteroblasty, reviewed in Chitwood and Sinha, 2016). As the leaf is the primary photosynthetic organ and therefore of paramount functional importance for plants, leaf shape has long been hypothesized to be adaptive (Givnish, 1979, 1995; Nicotra et al., 2011). However, leaf-shape variation between or within natural populations or closely related species can be controlled by genetic loci (Kidner and Umbreen, 2010) and thus may also be influenced by neutral processes, such as genetic drift and restricted gene flow. Yet few studies have detected genetic signatures of natural selection on leaf shape and related variation to putative adaptive function (Ferris, 2019; but see Bright and Rausher, 2008; Campitelli and Stinchcombe, 2013; Ferris and Willis, 2018; Richards et al., 2019), the adaptive feature of leaf shape still needs to be further elucidated.

The adaptive hypothesis of leaf shape is empirically based on the physiological and ecological consequences of leaf shape, although this type of evidence is typically correlative and indirect. Leaf-shape variation can affect the thickness of leaf boundary layer, which in turn affects thermoregulation (Givnish, 1979; Schuepp, 1993; Lambers et al., 2008). All else being equal, the thickness of a boundary layer increases with length from the windward edge, so that heat convection from small leaves is more rapid than from large leaves (Nobel, 2005). Likewise, leaf lobing can probably reduce the distance across the lamina, the rate of heat transfer is predicted to be greater in a lobed leaf than an unlobed leaf with equivalent area (Givnish, 1979; Nicotra et al., 2011). Compared with entire leaves, lobed and dissected leaves with reduced lobe/leaflet width can also have fewer minor veins and a lower ratio of mesophyll tissue to large, highly conductive veins, and thus lower hydraulic resistance, which can be advantageous in dry environments (Brodribb et al., 2010; Nicotra et al., 2011). Given these physiological effects, complex leaves (deeply divided or dissected and compound) are likely adaptive in particularly cold, hot, or dry habitats (Givnish, 1979; Ferris, 2019). Consistently, the global pattern clearly shows that extant plants with entire leaves are more frequent in subtropical and tropical, as well as frigid regions, while dissected leaves are often found in mid-latitude regions (reviewed in Chitwood and Sinha, 2016). Moreover, leaf shape may affect plant resistance to herbivores. Leaves of relatively palatable species may mimic the leaf shape of relatively distasteful species (Givnish, 1984; Brown and Lawton, 1991). Complex leaves may also gain advantage through isolating and/or limiting herbivorous damage to discrete areas, and making grazing less efficient for herbivores (Brown and Lawton, 1991; Niinemets et al., 2006).

From an evolutionary perspective, a classical approach to assess whether selection is responsible for phenotypic divergence within a species is to directly quantify fitness consequences of focal traits in different environments. This approach, typically performed by reciprocally transplanting individuals that are the product of controlled crosses into divergent natural field sites, can be very powerful (Rausher and Delph, 2015; Ferris, 2019). A few studies have detected natural selection on leaf shape with this approach, including *I. hederacea* (Bright and Rausher, 2008), the *Mimulus guttatus* species complex (Ferris and Willis, 2018), and the *Senecio lautus* species complex (Richards et al., 2019). However, this approach can have limitations. Because there may be substantial temporal and spatial variation in selective environments across species’ ranges, it can be difficult to extrapolate results from short-term, spatially restricted experiments, to an understanding of the forces shaping patterns of divergence across populations over generations (Hopkins et al., 2012). Moreover, this approach is only available for organisms that are amenable to field experiments in which fitness can be measured. For some plant species, e.g., perennial species, the total lifetime fitness can hardly be quantified.

A complementary approach that can circumvent some of the problems is the cline-fitting method, that is, to compare clinal patterns of focal traits to background patterns of differentiation at presumably neutral markers, as well as to environmental clines. If phenotypic patterns are explained by environmental variation rather than neutral population structure, then selection is likely at work (Ferris, 2019). Several studies have partially applied this approach to identify signatures of natural selection on leaf shape. They either revealed coincidence of phenotypic and geographic/macroclimatic clines without any genetic data (Wyatt and Antonovics, 1981; Gurevitch, 1988; Harris et al., 1998; Zhu et al., 2015; Alcántara-Ayala et al., 2020) or compared geographical leaf-shape cline with neutral genetic structure without any environmental association analysis (Brennan et al., 2009; Campitelli and Stinchcombe, 2013). Furthermore, both macrohabitat at coarse scale and microhabitat at fine scale can contribute to divergent selection thus to speciation (Coyne and Orr, 2004). Yet no universal relationship between leaf shape and broad-scale environmental variation has been found across taxa, which also suggests a potential role of natural selection at microhabitat level (Nicotra et al., 2011; Ferris, 2019). Therefore, an integrative study that combines geographic leaf-shape variation, neutral genetic structure, macrohabitat, and microhabitat analyses is likely to be more effective in understanding the drivers of leaf-shape variation.

**Figure 1:**
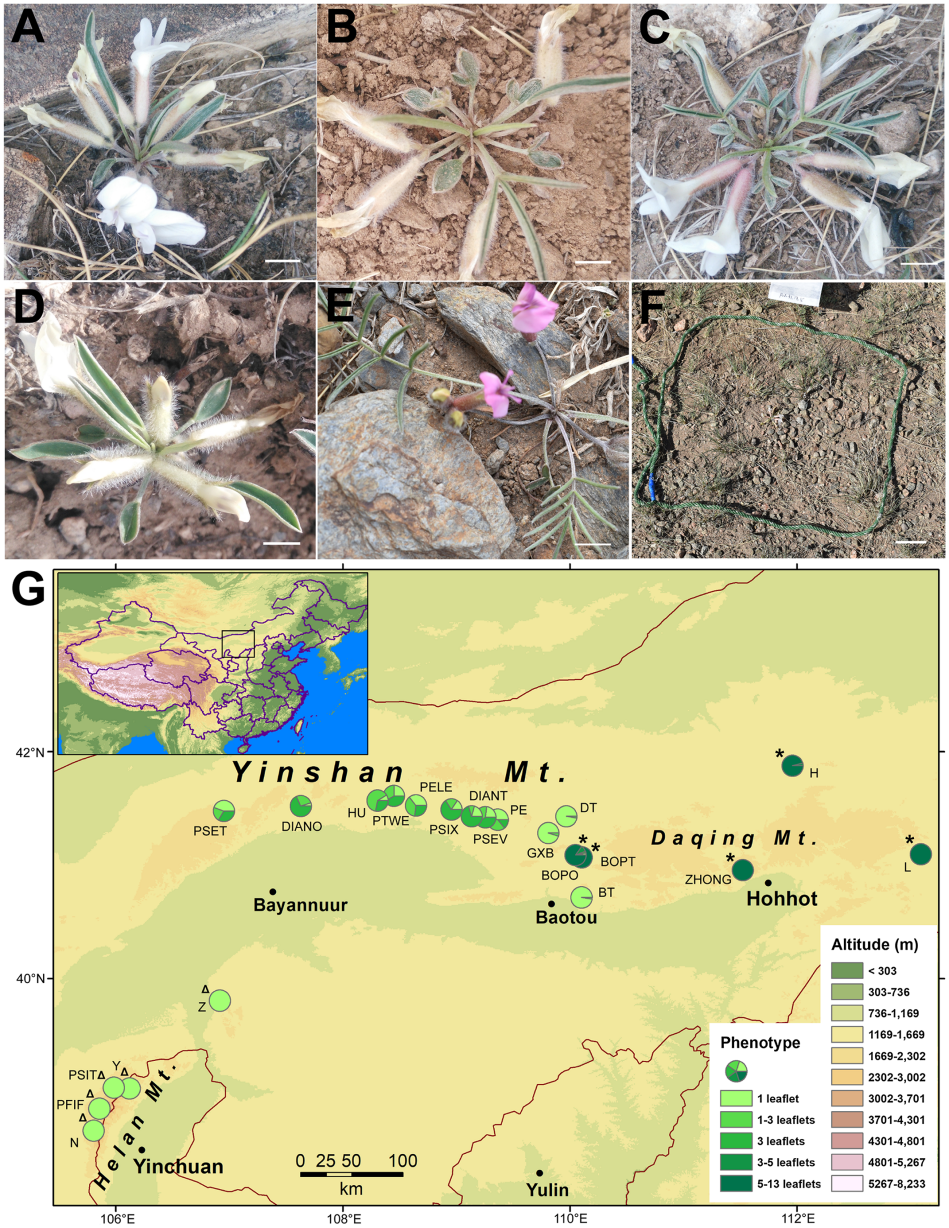
**(A–E)** Phenotypes of *Oxytropis diversifolia* (**A**, 1 leaflet; **B**, 1–3 leaflets; and **C**, 3 leaflets), *O. neimonggolica* (**D**, 1 leaflets), and *O. leptophylla* (**E**, 5–13 leaflets). Scale bars = 5 mm. **(F)** Example of a microhabitat plot (40 cm × 40 cm). Scale bar = 5 cm. **(G)** Map of northern China showing locations of 22 sampled *Oxytropis* populations. Each pie chart indicates the proportions of different leaf phenotypes in each population. Twelve populations are *O. diversifolia*; Δ five populations of *O. neimonggolica*; and ^*^ five populations of *O. leptophylla*.

*Oxytropis diversifolia* E. Peter is a perennial herb distributed across the steppes of Nei Mongol, China, which possesses an interesting pattern of intraspecific leaf-shape variation. Leaf shape in this species is polymorphic for leaflet production: Individuals can have leaves with only 1 leaflet (Figure 1A), 1 to 3 leaflets (Figure 1B), or 3 leaflets (Figure 1C). The three phenotypes can co-occur within populations without any spatial segregation. According to our population survey in three consecutive years (2016–2018), clinal variation for leaf shape is revealed, where eastern populations are composed chiefly of 1-leaflet phenotype, and western populations are highly polymorphic with phenotypes of 1–3 leaflets and 3 leaflets more frequent (Figure 1G). Moreover, two close relatives of *O. diversifolia* are also included in our study. One is *Oxytropis neimonggolica* C. W. Chang & Y. Z. Zhao, which is predominantly fixed for the 1-leaflet phenotype (Figure 1D) and allopatrically distributed in the Helan mountains (Figure 1G). The other is *Oxytropis leptophylla* (Pall.) DC., which is predominantly imparipinnate with 5–13 leaflets (Figure 1E) and parapatrically distributed to the east of *O. diversifolia* (Figure 1G). In the narrow zone of contact, the two species can hybridize with both pure and intermediate phenotypes co-existing, suggesting recent gene flow between the two species (Wang et al., unpubl. Res.). Having compound leaves are one of the main characteristics of Fabaceae and are evolutionary conserved in a phylogenetic context (Geeta et al., 2012). Leaf shape in *O. diversifolia* is highly polymorphic (1 leaflet, 1–3 leaflets, and 3 leaflets), while the two close relatives are either fixed for a phenotype of 1 leaflet (*O. neimonggolica*) or 5–13 leaflets (*O. leptophylla*), which can be used for comparison (predominantly monomorphic). Consequently, the three species form an ideal system in which to explore the evolutionary forces shaping the spatial pattern of leaf-shape variation.

Here, we use a comprehensive cline-fitting approach to assess the role of natural selection in explaining the spatial pattern of leaf shape in *O. diversifolia* as well as two close relatives. Specifically, we seek to address the following questions: (1) What is the spatial pattern of leaf-shape variation across the distribution range of *O. diversifolia* in Nei Mongol? Are leaf-morphological traits related to leaflet-blade size also differentiated among phenotypes? (2) Compared with *O. neimonggolica* and *O. leptophylla*, is there evidence of genetic differentiation and hierarchical population structure for *O. diversifolia* at neutral genetic markers (chloroplast DNA intergenic regions and nuclear microsatellites)? (3) Do microhabitat variables differ among different phenotypes? And/or do macroclimatic variables explain the clinal leaf-shape variation?

## Materials and Methods

### Study System

*Oxytropis diversifolia* (Fabaceae) is a diploid (2*n* = 16) perennial herb occurring in dry *Stipa* grasslands/semi-desert regions of Nei Mongol, China and Mongolia (Zhu et al., 2010). It is a typical xerophytic plant species with long roots (>50 cm) extending deep underground, while the aboveground part is very small (3–5 cm tall). It is acaulescent and the emerging leaves exhibit heteroblasty, with leaflet blades of early leaves being lanceolate-obovate to elliptic, while mature leaves are narrowly linear (Figures 1A–C). It can produce 1- or 2-flowered racemes and flowering occurs in April to May. Although automatic self-pollination does not occur, population genetic analysis suggests it is probably self-compatible (see results). It is bee-pollinated with an explosive pollination mechanism (e.g., Alemán et al., 2014). Livestock grazing can be observed in some of the populations (e.g., population DT and DIANT), but there does not seem to be an apparent difference of damage among phenotypes. Field-transplanting experiments in controlled conditions showed that leaf shape in this species is heritable (Supplementary Figures 1A–C), but the molecular genetic basis is still to be investigated.

*Oxytropis neimonggolica* is endemic to China and mainly found on sunny slopes of the Helan mountains (Chang and Zhao, 1981). The morphology of this species is similar to the 1-leaflet phenotype of *O. diversifolia* (Figure 1D). *O. leptophylla* is more widely distributed and can be found in northeastern China (Nei Mongol, Jilin, Hebei, Shanxi), Mongolia, and Russia (Zhu et al., 2010). The distinct morphological characteristics include imparipinnate leaves with 5–13 leaflets and a purple corolla (Figure 1E).

### Population Survey and Sampling

A total of 22 populations were surveyed in April and May from 2016 to 2018 (Figure 1G; Supplementary Table 1). We conducted a census of 12 and five populations for *O. diversifolia* and *O. neimonggolica*, respectively, covering their entire distributional range in Nei Mongol. For *O. leptophylla*, we sampled five populations in eastern Nei Mongol as representatives. In each population, leaf-phenotype surveys were performed by tallying leaf-phenotype counts (≤100 individuals), or along three to five transects spanning the length of the population (>100 individuals). The sampling effort, i.e., the number of transects and the distance between transects, depended on both population size and density.

We randomly sampled 20 (populations dominated by one phenotype) to 30 individuals (polymorphic populations) per population. Effort was made to distribute the sampling throughout the population and no neighboring plants were collected. In two extremely small populations Z and PSET, 11 and seven individuals were sampled, respectively. In polymorphic populations of *O. diversifolia*, effort was made to sample an equal number of individuals for each phenotype (Supplementary Table 7). For each individual, after taking the picture for microhabitat analysis, we collected several entire leaves (with the petiole and all leaflets, from both early leaves and mature leaves), immediately desiccated in silica gel, and stored in the dark at room temperature. In total 551 individuals were sampled, of which 321 were *O. diversifolia*, 128 were *O. neimonggolica*, and 102 were *O. leptophylla*.

### Measurements and Analyses of Leaf Morphology

Four leaf-morphological traits related with leaflet-blade size were measured by digital caliper for all 551 individuals: early leaf length, mature leaf length, early leaf width, and mature leaf width. Measurements were always done by the same experimenter (HY) to reduce sampling errors. The variation of these traits was then analyzed separately by performing linear mixed-effects models, with species or phenotype as a fixed effect and population nested within species as a random factor.

### DNA Extraction, Chloroplast DNA Sequencing, and Nuclear Microsatellite Genotyping

Genomic DNA was extracted from leaf tissue using the cetyltrimethyl ammonium bromide method (Doyle and Doyle, 1990). After preliminary universal primer screening, five cpDNA intergenic regions (*trn*T-*psb*D, *pet*N-*psb*M, *trn*S-*trn*G, *psb*E-*pet*L, and *rpl*16 intron; Supplementary Table 3) with good amplification and considerable nucleotide diversity were used for the large-scale survey of 237 individuals from 22 populations (Six to 17 individuals per population; Supplementary Table 4). Each PCR amplification was implemented in a 15 μl volume containing 7.5 μl 2 × TSINGKE Master mix (Tsingke Biological technology, Xi’an, Shaanxi, China), 1 μl of each primer at 10 μm, and 1 μl of diluted template DNA. PCR thermocycling conditions were 3 min of initial denaturation at 95°C, followed by 30 cycles of 30s at 94°C, 30s at the annealing temperature (Supplementary Table 3), 60s of elongation at 72°C, and ending with a 7 min extension at 72°C. Sequencing was conducted by Tsingke Biological technology on an ABI 3730xl DNA analyzer (Applied Biosystems, Foster City, CA, United States). All of the sequences were deposited in GenBank under accession numbers MN950995-MN952199 (including sequences of the four outgroup species, see below).

Nuclear DNA variation was characterized at 11 microsatellite loci for all 551 individuals (Supplementary Table 6). These microsatellites were retrieved from Wang et al. (2018) based on the reliability of amplification and the ease of scoring. The sequences of the primer pairs, PCR conditions, and the genotyping method were described in detail in Wang et al. (2018).

### Chloroplast DNA Sequence Analyses

#### Population Genetics

Sequences were aligned and edited using Geneious 9.0.2.^1^ The five fragments were concatenated for further analyses. Haplotypes were identified using DnaSP v6.12.01 (Rozas et al., 2017), with sites of nucleotide substitutions considered only. Several genetic diversity parameters, including number of segregating sites (*S*), number of haplotypes (*h*), haplotype diversity (*Hd*), and nucleotide diversity (*π*; Nei, 1987), were calculated for each population and also at the species level using DnaSP. To test whether the observed amount of genetic diversity deviates from neutral expectations, we estimated Tajima’s *D* (Tajima, 1989), Fu and Li’s D^*^ and F^*^ (Fu and Li, 1993), and assessed the significance of these statistics in DnaSP with default settings.

To explore whether genetic diversity at the population level was different among species, we fitted a linear model with those genetic diversity parameters in each population as response variables, the number of individuals sampled, population size (with the basic assumption that genetic diversity increases with both of them), and species identity as explanatory variables. The significant contribution of each explanatory variable was tested using the stepwise multiple regression procedure.

To examine how nucleotide variation is partitioned among species, as well as among populations within *O. diversifolia*, we investigated population structure using the spatial analysis of molecular variance (SAMOVA, Dupanloup et al., 2012) implemented by SPADS 1.0 (Dellicour and Mardulyn, 2014). We ran 10 independent replicates testing for 2–20 groups (*K*), and the number of iterations was set to 10,000.

#### Phylogenetic Relationship

The phylogenetic relationship among haplotypes was reconstructed based on maximum parsimony (MP) and Bayesian inference (BI). We used four species collected in Nei Mongol (*O. aciphylla*, *O. bicolor*, *O. racemosa*, and *O. squammulosa*) as outgroups. For all the analyses, we considered sites of nucleotide substitutions only. PAUP^*^ 4.0b10 (Swofford, 2002) was used to conduct the MP analyses with the following settings: heuristic search, TBR branch-swapping, 1,000 bootstrap replicates, random addition sequence with 10 replicates, and a maximum of 1,000 trees saved per round. jModelTest v2.1.7 (Darriba et al., 2012) was used to choose the best-fit nucleotide substitution model following the Bayesian Information Criterion (BIC). The Bayesian inference was performed using MrBayes v3.2.5 (Ronquist and Huelsenbeck, 2003; Ronquist et al., 2012). Two independent analyses starting from different random trees with three heated and one cold chain were run for 10,000,000 generations, and trees were sampled every 1,000 generations (10,000 trees sampled in total). The first 2,500 trees (25%) were discarded as burn-in. Tree visualization was achieved in FigTree v1.4.3.^2^ A statistical parsimony network based on Median Joining method (Bandelt et al., 1999) was constructed and visualized using PopArt 1.7 (Leigh and Bryant, 2015).

### Nuclear Microsatellite Analyses

#### Microsatellite Polymorphism and Genetic Diversity

We first tested genotypic linkage disequilibrium between each pair of loci within each population, using the G test available in GENEPOP 4.7 (Rousset, 2008). Multiple tests were then corrected using Benjamini–Hochberg correction (Benjamini and Hochberg, 1995). We also used exact tests implemented by GENEPOP to test for departure from Hardy–Weinberg equilibrium (Rousset and Raymond, 1995). Because heterozygote deficiency was recorded for almost all loci across populations, we tested large allele dropout and stuttering using Micro-Checker 2.2.3 (van Oosterhout et al., 2004) and estimated null allele frequencies using the EM algorithm with the program FreeNA (Chapuis and Estoup, 2007). Several genetic diversity parameters, including number of alleles (*N*_a_), observed heterozygosity (*H*_o_), and unbiased expected heterozygosity (*H*_e_) and *F*_IS_, were calculated using GenAlEx version 6.503 (Peakall and Smouse, 2006, 2012). Linear models were fitted as described in the “Chloroplast DNA Sequence Analyses” section.

#### Genetic Differentiation and Isolation by Distance

Pairwise *F*_ST_ estimates were calculated following Weir and Cockerham (1984) in GENEPOP, and tests of the genotypic differentiation among populations were performed using the exact G test provided by GENEPOP. To test for isolation by distance (IBD), we regressed linearized genetic differentiation between populations, measured as *F*_ST_/(1 − *F*_ST_; Rousset, 1997), and geographic distances using Mantel tests with 10,000 permutations.

#### Population Structure

We used the Bayesian clustering program STRUCTURE 2.3.4 (Pritchard et al., 2000) to probabilistically assign all individuals to a varying number of clusters (*K*). We used an admixture model with independent allele frequencies and ran 10 replicates for each value of *K* = 1–10 with a burn-in of 10,000 Markov Chain Monte Carlo (MCMC) steps followed by 50,000 iterations. The best *K* was determined using the method of Pritchard et al. (2000) and Evanno et al. (2005) in CLUMPAK (Kopelman et al., 2015). The individual cluster assignments were determined and visualized also using CLUMPAK. Additionally, we used the program InStruct (Gao et al., 2007), which is based on a similar algorithm to STRUCTURE but allows for partial self-fertilization or inbreeding, to verify our results.

### Micro- and Macrohabitat Analyses

#### Microhabitat Differentiation

To detect any potential effect of abiotic environment on the observed pattern of leaf-phenotypic differentiation, we collected data of different niche variables separated into microhabitat and macrohabitat axes, according to the hierarchical scale at which the variables were measured. The microhabitat axes captured habitat attributes at the local scale of individuals within populations. For each individual sampled, we surveyed a 40 cm × 40 cm square plot, of which the edge length was about four times the focal plant height (plant height < 10 cm; Figure 1F). We measured slope (by geologic compass), the percent of total vegetation cover, the percent of rocky ground (rock diameter > 0.2 cm), and the percent of bare ground (sand and soil). The latter three were scored on pictures by eye, and always done by the same experimenter (HW). In total, we collected microhabitat data for 438 individuals in 18 populations (Supplementary Table 7). We then conducted linear mixed-effects models on the four variables as described in the “Measurements and Analyses of Leaf Morphology” section.

#### Macrohabitat Differentiation

The macrohabitat axes of bioclimatic data were retrieved based on field sampling localities at the coarse scale (22 records; Supplementary Table 1). Nineteen contemporary bioclimatic variables of the time period 1970–2000 at 30-s resolution were downloaded from WorldClim^3^ (Hijmans et al., 2005). We conducted PCA on the 19 variables, and then removed highly correlated variables while keeping relatively orthogonal variables important to plant physiology and phenology, and hence geographical distribution for further analyses: annual mean temperature (Bio01), mean diurnal range (Bio02), isothermality (Bio03), temperature seasonality (Bio04), annual precipitation (Bio12), and precipitation seasonality (Bio15). We also extracted aridity index for those localities from the Global Aridity Index database (at 30-s resolution of the time period 1970–2000; Trabucco and Zomer, 2018). Aridity index is calculated as mean annual precipitation divided by mean annual potential evapotranspiration, which can be a better index of relative moisture supply based on the prevailing climate instead of annual precipitation.

According to the leaf ecophysiological theory, leaf lobation/dissection can affect leaf thermoregulation and hydraulic efficiency mainly through reduction in effective leaf size (see Introduction). For *O. diversifolia*, in order to explain variation of the four leaf-morphological traits measured with macroclimatic variables, two strategies were used as: population-based model and individual-based model. Firstly, at the population level (*N* = 12), we calculated the weight mean of leaflet-blade size indicator for each population as response variable, i.e., (mean X of 1 leaflet) × (proportion of 1 leaflet) + (mean X of 1–3 leaflets) × (proportion of 1–3 leaflets) + (mean X of 3 leaflets) × (proportion of 3 leaflets), in which X can be early leaf length, mature leaf length, early leaf width, or mature leaf width. We then constructed a linear model including six macroclimatic variables and altitude as explanatory variables (i.e., full model; the null model had no explanatory variables but only mean value of the response variable as intercept), and performed spatial autocorrelation tests for the simulated residuals (1,000 permutations) from both the null model and the full model. For early/mature leaf width, although residuals from the null model showed spatial autocorrelation (Moran’s index = 0.12/0.092, *p* = 0.037/0.067), residuals from the full model showed no spatial autocorrelation (*p* = 0.33/0.99), which indicates the spatial autocorrelation of early/mature leaf width can be explained by those variables. Next, a stepwise multiple regression analysis was performed. Data were scaled prior to analyses. Secondly, at the individual level, we randomly selected maximum individuals in each population according to leaf-shape phenotype frequency (*N* = 231) and then performed linear mixed-effects models with spatially autocorrelated random effects considered (longitude and latitude coordinates as spatial information). The explanatory variables (fixed effects) included six macroclimatic variables, altitude, and STRUCTURE admixture proportion (green cluster value in *K* = 4). The significance of each variable was tested through likelihood-ratio test (for details see Imbert, 2020).

All of the statistical analyses were performed using R software version 3.5.2 (R Core team, 2018). PCA was performed using the “FactoMineR” library (Lê et al., 2008). General linear mixed-effects models were fitted using the “lmer” function from the “lme4” library (Bates et al., 2015). Linear mixed-effects models with spatially autocorrelated random effects considered were fitted using the “fitme” function from the “spaMM” library (Rousset and Ferdy, 2014). Spatial autocorrelation analyses were performed using the “DHARMa” library (Hartig, 2020). Mantel tests were performed with the “ecodist” library (Goslee and Urban, 2007).

## Results

### Spatial Pattern of Leaf-Shape Distribution

For *O. diversifolia*, three populations in the east were dominated by the 1-leaflet phenotype (>95.0%) coupled with the phenotype of 1–3 leaflets only, while the three phenotypes co-occurred in the remaining nine populations (1 leaflet, 1–3 leaflets, and 3 leaflets; Figure 1G; Supplementary Table 1). As a whole, the range and mean of proportions of three phenotypes in *O. diversifolia* populations were 5.3–97.6% (43.1%), 2.4–63.1% (24.4%), and 0–70.2% (32.4%), respectively. For *O. neimonggolica*, four out of five populations were monomorphic with 1-leaflet phenotype (Figure 1G), and the only exception was one individual with 1–3 leaflets in the population PFIF (0.7%; Supplementary Table 1). The five populations of *O. leptophylla* were predominantly monomorphic with the phenotype of 5–13 leaflets, and exceptions were found in BOPO (one with 1 leaflet, one with 3 leaflets, and three with 3–5 leaflets; 15.1%) and H (one with 3–5 leaflets; 4.0%; Figure 1G; Supplementary Table 1).

For 12 populations of *O. diversifolia*, the 1-leaflet proportion showed significant spatial autocorrelation (Moran’s index = 0.22, *p* = 0.0018). Autocorrelation was also revealed for the proportion of 1–3 leaflets (Moran’s index = 0.13, *p* = 0.031) and marginally detected for the proportion of 3 leaflets (Moran’s index = 0.094, *p* = 0.065). Similarly, a significant positive relationship was found between the difference in 1-leaflet proportion (Euclidean distance) and the geographical distance (Mantel test, *r* = 0.43, *p* = 0.013), but this relationship was not detected for the difference in the proportion of 1–3 leaflets or for 3 leaflets (*p* > 0.10). Significant correlations were also observed between 1-leaflet proportion and longitude (Spearman’s rank test, *r* = 0.70, *N* = 12, *p* = 0.015), as well as latitude (*r* = −0.62, *p* = 0.035). The proportion of 1–3 leaflets was marginally correlated with longitude (*r* = −0.56, *p* = 0.063) and latitude (*r* = 0.57, *p* = 0.059), while the proportion of 3 leaflets was significantly correlated with longitude (*r* = −0.63, *p* = 0.029), but not with latitude (*p* = 0.12). No correlation was detected between phenotype proportion and altitude (*p* > 0.10).

### Inter- and Intraspecies Variation of Leaf Morphology

**Figure 2:**
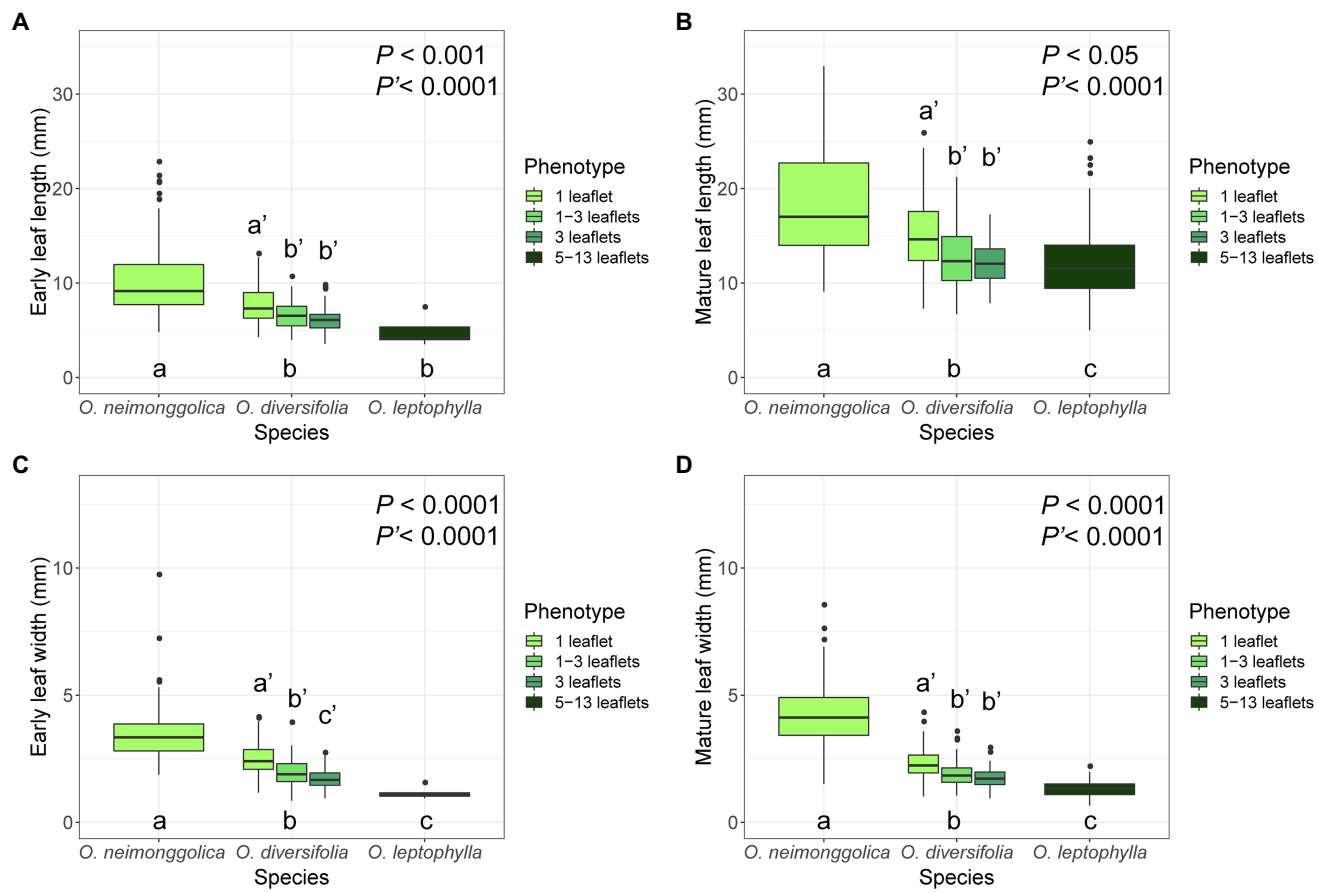
Boxplots of four leaf-morphological traits measured showing inter- and intraspecific leaflet-blade size differentiation. **(A)** early leaf length; **(B)** mature leaf length; **(C)** early leaf width; and **(D)** mature leaf width. Values of *P* indicate the significance of differences among species (*P*) or among phenotypes of *O. diversifolia* (*P′*) in linear mixed-effects models, and different letters indicate significant differences of pairwise comparisons (*p* < 0.05, Tukey’s HSD test).

Concerning the four leaf-morphological traits related with leaflet-blade size measured, we found that they were all significantly differentiated among the three species (*p* < 0.05 in all the cases), with *O. neimonggolica* > *O. diversifolia* > *O. leptophylla* (Figure 2; Supplementary Table 2). Similarly, for the intraspecies analysis of *O. diversifolia*, the leaf-shape phenotype effect was also significant for the four traits measured (*p* < 0.0001 in all the cases), with higher value of 1-leaflet phenotype than phenotypes of 1–3 leaflets and 3-leaflets (Figure 2; Supplementary Table 2). Overall, the inter- and intraspecific morphological differentiation exhibited a similar trend, the leaflet blade was smaller if the species/phenotype had more leaflets (Figure 2).

### cpDNA Sequence Variation

#### cpDNA Haplotypes and Genetic Diversity

**Figure 3:**
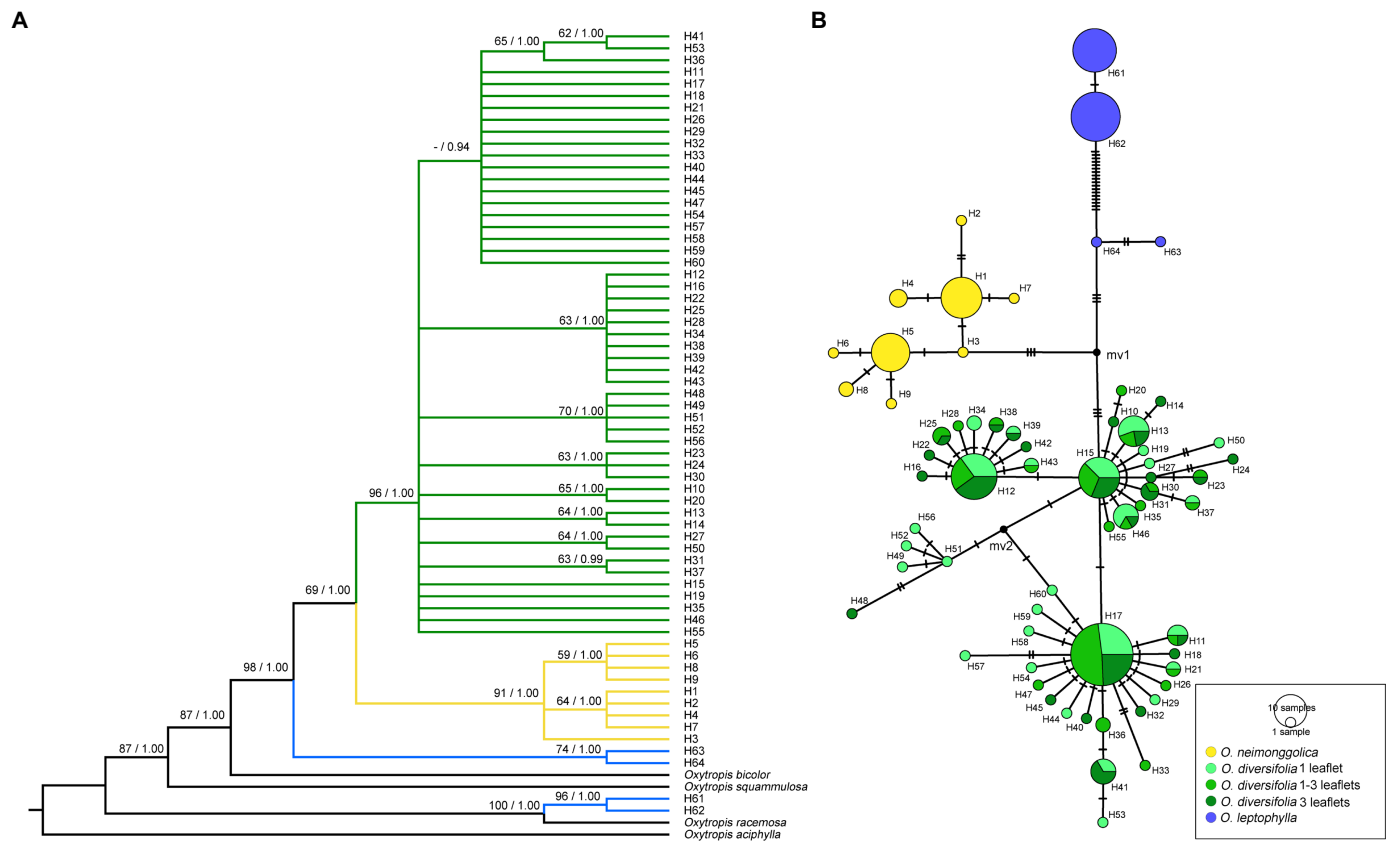
Phylogenetic relationships based on cpDNA haplotypes. **(A)** The Bayesian tree topology of 64 cpDNA haplotypes detected in the three *Oxytropis* species. Numbers above the branches are MP bootstrap support values (left) and Bayesian posterior probability (right). **(B)** Network-derived genealogical relationships of the 64 haplotypes.

The concatenated cpDNA dataset had a total alignment length of 3,849 bp. Across all 237 sequences, 64 haplotypes were identified, characterized by 88 variable sites, of which 49 were parsimony informative. The three species were fixed for distinct haplotypes, with H1–H9 in *O. neimonggolica*, H10–H60 in *O. diversifolia*, and H61–H64 in *O. leptophylla* (Figure 3). In *O. diversifolia*, the number of haplotypes (*h*) per population ranged from four (BT) to 11 (PTWE; Supplementary Table 4). Three most frequent haplotypes (H12, H15, and H17; Figure 3B) accounted for 47.4% of the samples, which could be found in 5, 9, and 10 out of 12 populations, respectively (Supplementary Figure 2). The majority of haplotypes (72.5%, 37 out of 51) was fixed in one population (private haplotypes, *h*_p_), ranged from one to six per population (Supplementary Table 4). We found that Tajima’s *D*, Fu and Li’s D^*^ and F^*^ did not deviate from neutrality for any individual population (Supplementary Table 4). However, these values were significantly negative for all populations combined in *O. diversifolia* (*p* < 0.01; Table 1A).

**Table 1:** Descriptive statistics of genetic variability based on cpDNA and 11 nuclear microsatellite loci.

	*O. neimonggolica*	*O. diversifolia*	*O. leptophylla*	Value of *p*
	(5 populations)	(12 populations)	(5 populations)	
(A) cpDNA				
*S*	2.4(0.548)	9.1(3.40)	5(8.94)	0.30
*h*	2.8(0.447)^a^	7.8(2.12)^b^	2.4(0.894)^a^	<0.01
*h*_p_	1.4(0.548)^ab^	3.1(1.56)^a^	0.4(0.894)^b^	0.08
*Hd*	0.536(0.182)^a^	0.880(0.0736)^b^	0.523(0.132)^a^	<0.001
*π*	0.00025(0.00010)	0.00061(0.00020)	0.00054(0.00092)	0.42
Tajima’s *D*	−0.838 ns	**−2.339** ^**^	−1.779 ns	
Fu and Li’s D^*^	−1.773 ns	**−5.798** ^**^	1.008 ns	
Fu and Li’s F^*^	−1.733 ns	**−5.187** ^**^	0.074 ns	
(B) Nuclear microsatellites				
*N*_a_	10.7(2.13)^a^	10.8(2.26)^a^	7.3(0.606)^b^	<0.01
*H*_o_	0.536(0.0225)^ab^	0.578(0.0331)^a^	0.495(0.0379)^b^	<0.01
*H*_e_	0.752(0.0524)^a^	0.821(0.0212)^b^	0.708(0.0256)^a^	<0.0001
*F*_IS_	0.271(0.0194)	0.296(0.0318)	0.304(0.0403)	0.21

cp DNA: S, number of segregating sites; h, number of haplotypes; h_p_, number of private haplotypes (i.e., haplotypes that are fixed in only one population); Hd, haplotype diversity; and π, nucleotide diversity. Nuclear microsatellites: N_a_, number of alleles; H_o_, observed heterozygosity; H_e_, unbiased expected heterozygosity; and F_IS_, fixation index. For those genetic diversity parameters, data are presented as mean (s.d.) averaged across populations; values of p indicate the significance of differences among species in linear models. a and b indicate significant differences of pairwise comparisons (p < 0.05, Tukey’s HSD test). ns, not significant, p > 0.10;

** *p < 0.01.*

At the species level, the genetic diversity parameters (*S*, *h*, *h*_p_, and *Hd*) were highest in *O. diversifolia* (54, 51, 37, and 0.910, respectively; Supplementary Table 4). At the population level, the species effect was still significant for estimates of *h* and *Hd*, while marginally significant for the estimation of *h*_p_ (Table 1A).

#### Population Structure

Spatial analysis of molecular variance results indicated that *F*_CT_ reached a plateau when *K* = 3 (Supplementary Figure 3), in congruence with the species delimitation. When *K =* 4–7, further population segregations could be found in *O. neimonggolica* and *O. leptophylla*, but not in *O. diversifolia* (Supplementary Table 5).

#### Phylogenetic Relationship

The phylogenetic analyses based on BI and MP methods yielded largely congruent topologies (Figure 3A). Two well-supported sister clades were revealed, corresponding to *O. neimonggolica* (H1–H9) and *O. diversifolia* (H10–H60), respectively. The rare haplotypes in *O. leptophylla* (H63 and H64) had a close relationship with the former two major clades, while the main haplotypes (H61 and H62) were close to the outgroup species *O. racemosa*.

The relationships of those haplotypes were further illustrated using parsimony network (Figure 3B). We revealed that haplotypes of *O. diversifolia* could be categorized into four haplogroups, which were derived from three main haplotypes (H12, H15, and H17) and H51. H15- and H17-derived haplogroups could be found in all 12 populations (Supplementary Figure 2). The proportions of three leaf-shape phenotypes in three main haplotypes/haplogroups were not significantly different from the expected proportion (*Χ*^2^ = 0.0042–4.73, df = 2, *p* > 0.05). Consistently, the phenotypes and haplotypes/haplogroups were not significantly associated (Pearson’s *Chi*-squared test, *Χ*^2^ = 3.72/2.47, df = 4, *p* > 0.05).

### Nuclear Microsatellite Variation

#### Microsatellite Polymorphism and Genetic Diversity

The allelic diversity was highly variable among 11 loci genotyped, with the total number of alleles ranging from 10 to 47, and the average number of alleles (*N*_a_) per population varying from 5.5 to 15.3 (Supplementary Table 6). Thirty-three out of 1,210 (2.7%) within-population genotypic disequilibria were significant at the 5% level after Benjamini–Hochberg correction but were not specific for any pairwise loci or population. Significant heterozygote deficits were observed in 155 out of 242 locus-specific tests. Consistently, in all 22 populations, *F*_IS_ across loci was significantly higher than expected and ranged from 0.23 to 0.35 (Supplementary Table 7). We found no evidence of large allele dropout and the genotyping error of stuttering was scarce, but the presence of null alleles at all loci was suggested by Micro-Checker. The null allele frequencies were low to moderate in most loci (0.038–0.18), but locus N745892 and N2717495 exhibited a high level of null alleles (0.21 and 0.23, respectively). Excluding these two loci decreased the *F*_IS_ values (0.12–0.30), but all values were still significantly larger than zero (data not shown). Thus, it seems these two loci were not the only cause of heterozygosity deficits, and the systematic pattern of heterozygote deficiency found in our study is likely due to the self-compatibility and/or inbreeding. We then retained all 11 loci to calculate genetic diversity indices and estimated genetic structure.

**Figure 4:**
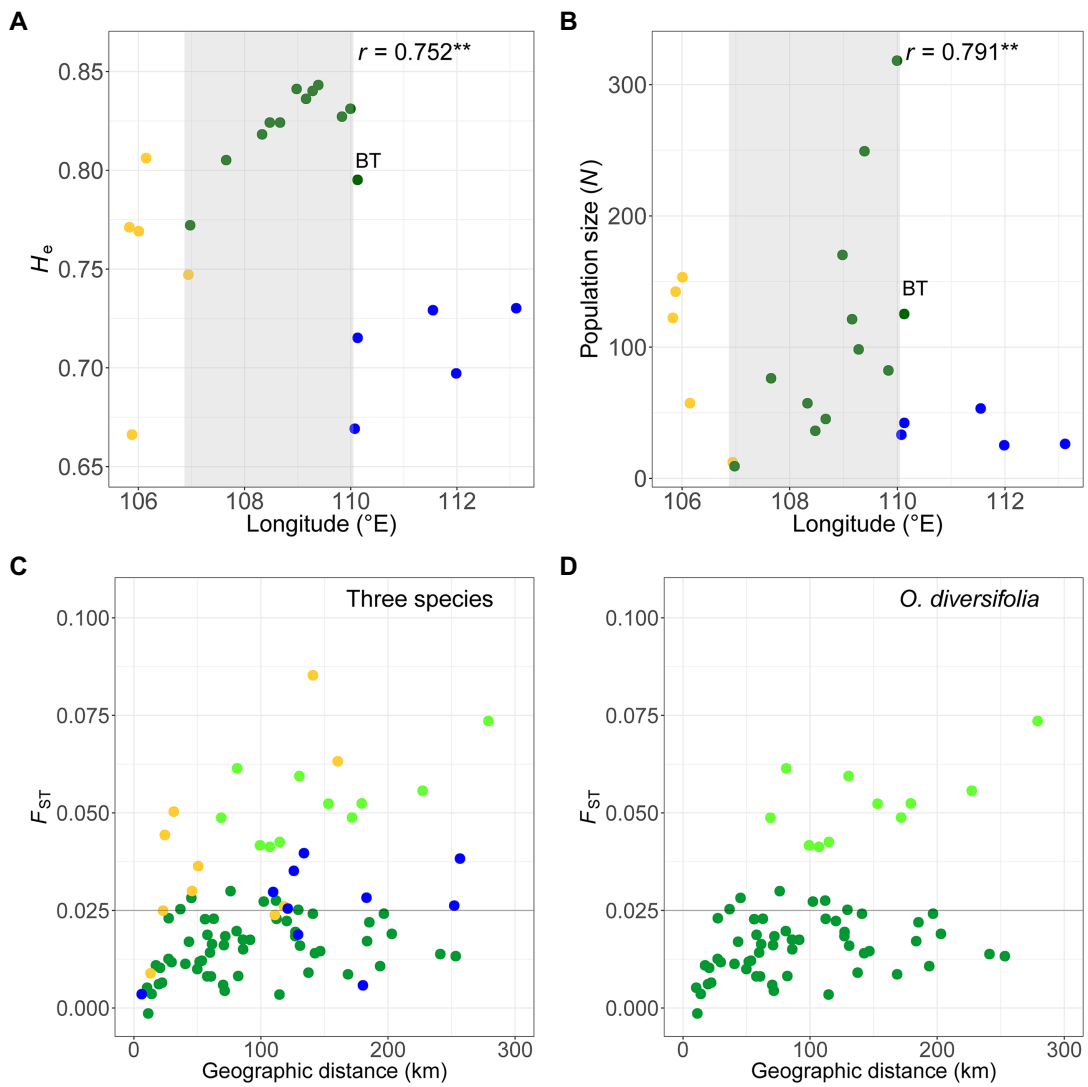
**(A,B)** Scatterplots of **(A)** the unbiased expected heterozygosity (*H*_e_) based on microsatellite dataset and **(B)** the estimated population size (i.e., the total number of individuals in the population) against longitude in 22 populations. Light grey shaded parts indicate a significant cline in *O. diversifolia* excluding population BT (coefficients of Spearman’s rank test were shown, ^**^p < 0.01). **(C,D)** Scatterplots of pairwise *F*_ST_ calculated from microsatellite dataset against geographic distances (km). **(C)** Pairwise combinations of populations in the three species. **(D)** Pairwise combinations of populations in *O. diversifolia*. Light green circles represent the pairwise combinations between BT, a 1-leaflet-dominated population, and the other populations of *O. diversifolia*. Yellow circles, *O. neimonggolica*; green circles, *O. diversifolia*; and blue circles, *O. leptophylla*. Forty-nine out of 55 *F*_ST_ values of *O. diversifolia* were below 0.025, while only three out of 10 *F*_ST_ values of *O. neimonggolica*/*O. leptophylla* were below 0.025 (the horizonal line of *F*_ST_ = 0.025 is highlighted in C and D)

At the species level, the genetic diversity parameters (*N*_a_, *H*_o_, and *H*_e_) across loci were highest in *O. diversifolia* (22.7, 0.583, and 0.845, respectively; Supplementary Table 7). Similarly, at the population level, the species effect was also significant for estimates of *N*_a_, *H*_o_, and *H*_e_ (Table 1B). Moreover, in *O. diversifolia* when excluding population BT with low genetic diversity (Supplementary Table 7) but high genetic differentiation with other populations (see results below), *H*_e_ showed a significant correlation with longitude (Spearman’s rank test, *r* = 0.75, *N* = 11, *p* = 0.0076), that is, *H*_e_ decreased from east to west (Figure 4A). We also found that the population size decreased westward as well (*r* = 0.79, *p* = 0.0061; Figure 4B).

#### Genetic Differentiation and Isolation by Distance

Pairwise *F*_ST_ (22 populations and 231 pairs) ranged from 0 to 0.20: 217 values of *p* were significantly different from 0 at the 5% level (exact G test). *F*_ST_ values between pairwise populations of different species were generally high, with *O. leptophylla* – *O. neimonggolica* (*F*_ST_ = 0.16 ± 0.021, mean ± s.d.) > *O. leptophylla* – *O. diversifolia* (*F*_ST_ = 0.11 ± 0.014) > *O. diversifolia* – *O. neimonggolica* (*F*_ST_ = 0.083 ± 0.022). By contrast, the *F*_ST_ values between pairwise populations of the same species were relatively low (Figure 4C), with 0.021 ± 0.016 in *O. diversifolia*, 0.039 ± 0.022 in *O. neimonggolica*, and 0.025 ± 0.012 in *O. leptophylla*, respectively. It is worth noting that, in *O. diversifolia*, most of the largest *F*_ST_ values were associated with a single population, the 1-leaflet-dominant population BT (*F*_ST_ = 0.053 ± 0.0098), indicating a genetic discontinuity between this population and the other populations (Figure 4D). When excluding BT, the *F*_ST_ values in *O. diversifolia* were even lower (0.015 ± 0.0073), and 49 out of 55 *F*_ST_ values were below 0.025. A marginally significant IBD pattern was obtained in *O. diversifolia* (Mantel test, *r* = 0.43, *p* = 0.071; Figure 4D) and *O. neimonggolica* (*r* = 0.54, *p* = 0.084), but not in *O. leptophylla* (*p* = 0.32). When removing BT from the *O. diversifolia* dataset, no IBD was detected (*r* = 0.25, *p* = 0.14; Figure 4D).

#### Population Structure

**Figure 5:**
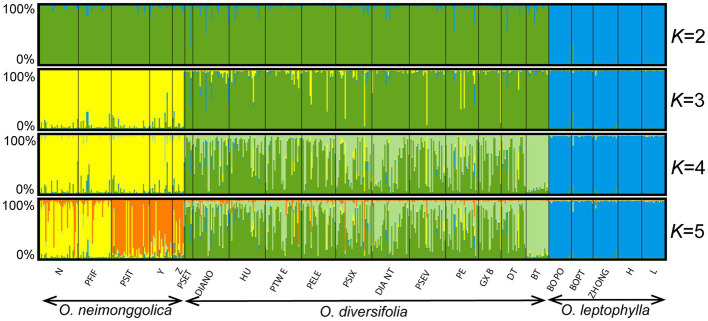
STRUCTURE results for 22 *Oxytropis* populations based on microsatellite dataset (*K* = 2–5). The small black lines separate populations, named at the bottom of the graph. Each individual is a small bar with color coded according to probability of clustering in a particular group. Populations are ordered west to east within each species.

According to the STRUCTURE analysis, Δ*K* was highest when *K* = 3 (Supplementary Figure 4A), but Prob(*K*) estimation indicated that the optimal value for *K* was 5 (Supplementary Figure 4B). We showed the results with *K* = 2–5 (Figure 5). For *K* = 2, individuals from *O. leptophylla* populations were predominantly assigned to the blue cluster, while the ones from *O. neimonggolica* and *O. diversifolia* were assigned to the green cluster. For *K* = 3, the assignments of individuals were in good congruence with the three species delimitation, although nine individuals (four of 1 leaflet, two of 1–3 leaflets, and three of 3 leaflets) of *O. diversifolia* had probabilities greater than 0.50 of being assigned to the yellow *O. neimonggolica* cluster. For *K* = 4, the individuals of *O. diversifolia* populations were further partitioned into different clusters. The likelihood of individuals from population BT being assigned to the light green cluster was 0.92 ± 0.041 (mean ± s.d.). Taken all the 12 populations together, individuals of the three phenotypes did not differ substantially in being assigned to the green cluster (0.50 ± 0.35 for 1 leaflet, 0.54 ± 0.33 for 1–3 leaflets, and 0.57 ± 0.33 for 3 leaflets; one-way ANOVA, *p* = 0.25). We also tested for correlation between admixture proportions (green cluster) and four leaf-morphological traits measured, and all the tests were not significant (Pearson’s correlation test, *N* = 302–311, *p* = 0.26–0.75) except for mature leaf width with a weak correlation (*r* = −0.13, *N* = 311, *p* = 0.020). After controlling for latitude and longitude, the correlation was not significant (Pearson’s partial correlation test, *p* = 0.48). Finally, the analysis of *K* = 5 exhibited further partitioning of populations from *O. neimonggolica*, with population N and PFIF differed from PSIT, Y, and Z in average likelihood of individuals being assigned to the yellow cluster (0.81 ± 0.24 vs. 0.17 ± 0.26). The results obtained from the InStruct program were largely congruent with the STRUCTURE results (Supplementary Figure 5).

### Micro- and Macrohabitat Differentiation

#### Microhabitat Differentiation

At the species level, we found that the values of slope (°) in *O. diversifolia* (4.94 ± 3.64, mean ± s.d.) were significantly lower than those of the other two species (14.9 ± 11.2 in *O. neimonggolica* and 13.1 ± 9.94 in *O. leptophylla*, respectively; *p* = 0.0055; Supplementary Table 8). However, the percent of total vegetation cover, the percent of rocky ground, and the percent of bare ground did not differ among species (*p* = 0.16–0.82; Supplementary Table 8). Similarly, in *O. diversifolia*, the leaf-shape phenotype effect was marginally significant for slope (6.28 ± 4.77 for 1-leaflet phenotype, 3.89 ± 2.27 for 1–3 leaflets, and 4.41 ± 2.58 for 3 leaflets; *p* = 0.076; Supplementary Table 8), but not for the other three variables (*p* = 0.19–0.71; Supplementary Table 8).

#### Macrohabitat Differentiation

**Figure 6:**
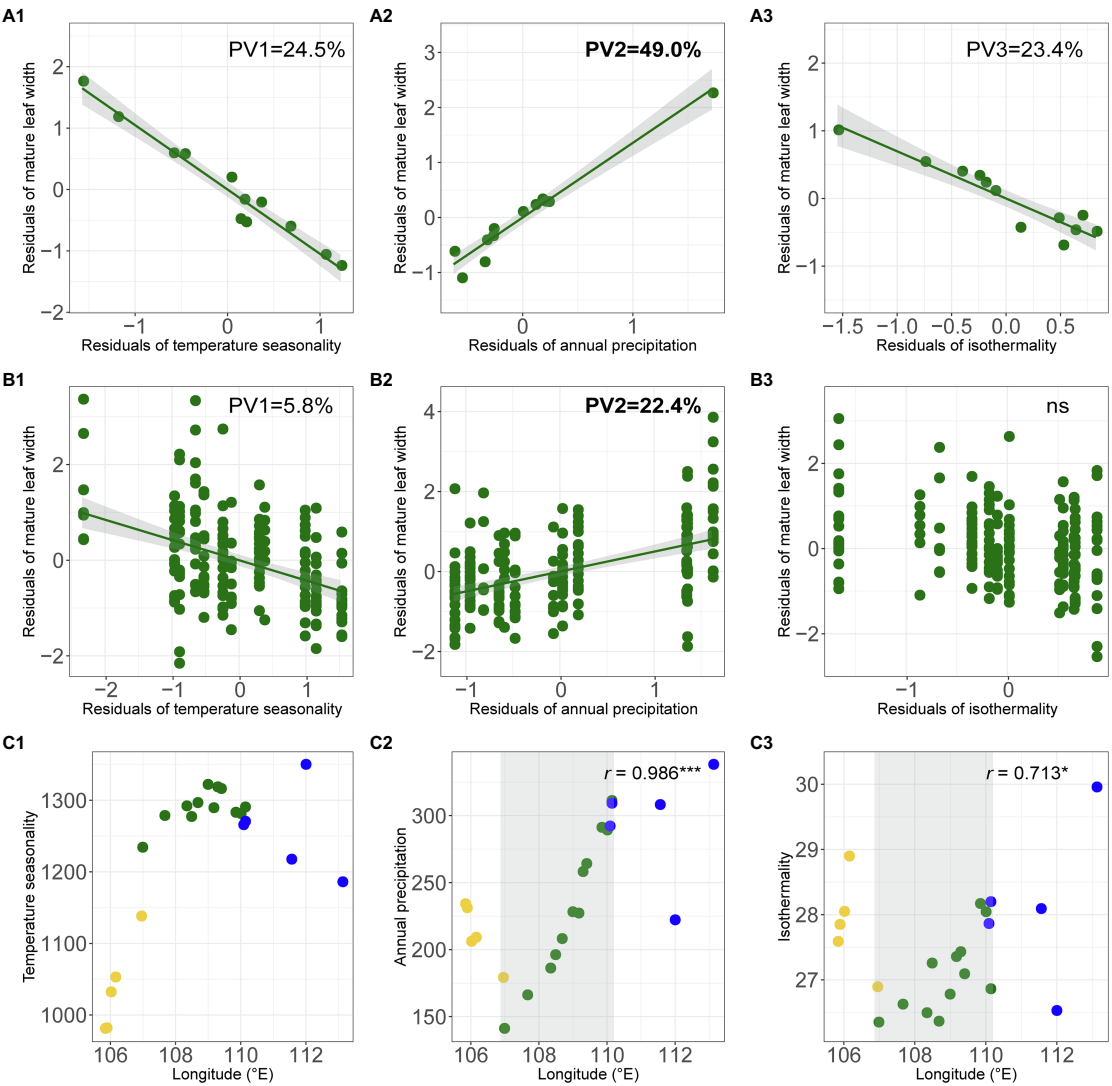
**(A1–B3)** Partial regression plots showing effects of predictive macroclimatic variables on mature leaf width of *O. diversifolia*. **(A1–A3)** Population-based model (*N* = 12); **(B1–B3)** Individual-based model (*N* = 213). PV1, percentage of variation explained by temperature seasonality; PV2, percentage of variation explained by annual precipitation; and PV3, percentage of variation explained by isothermality. **(C1–C3)** Scatterplots of temperature seasonality, annual precipitation, and isothermality against longitude in 22 *Oxytropis* populations. Yellow circles, *O. neimonggolica*; green circles, *O. diversifolia*; and blue circles, *O. leptophylla*. Light grey shaded parts indicate a significant cline in *O. diversifolia* (coefficients of Spearman’s rank test were shown, ^*^p < 0.05; ^***^p < 0.001).

Across the whole range of *O. diversifolia*, the population-based model revealed that the multiple regressions of weight means of four leaflet-blade size related traits and selected bioclimatic variables were significant with high predictive power (Multiple *R*^2^ = 0.788–0.969, *p* < 0.01 in all the cases; Table 2). The best two predictors for weight mean of early/mature leaf length were temperature seasonality and isothermality, which explained 42.3/35.3% and 41.1/38.5% of the total variance, respectively, followed by annual precipitation that explained 10.3/5.0% (Table 2A). However, the best predictor of early/mature leaf width was annual precipitation that alone explained 52.8/49.0% of the total variance, followed by temperature seasonality and isothermality (annual mean temperature for early leaf width), which explained 4.3/24.5% and 25.6/23.4%, respectively (Table 2A; Figures 6A1–A3). The remaining variables were not significant predictors (*p* = 0.13–0.99).

**Table 2:** Results of macroclimatic-association analysis for *O. diversifolia* based on multiple linear regressions.

	Temperature seasonality	PV1(%)	Annual precipitation	PV2(%)	Isothermality^a^	PV3(%)	Multiple *R*^2^	*F*-statistic	Value of *p*
(A) Population-based model (*N* = 12)									
Early leaf length	**−1.052** ^***^	**42.3**	1.108^***^	10.3	**−0.923** ^***^	**41.1**	0.937	*F*_3,8_ = 39.97	<0.0001
Mature leaf length	**−0.936** ^**^	**35.3**	0.973^**^	5.0	**−0.893** ^**^	**38.5**	0.788	*F*_3,8_ = 9.947	<0.01
Early leaf width	−0.700^**^	4.3	**1.160** ^***^	**52.8**	0.587^**^	25.6	0.827	*F*_3,8_ = 12.75	<0.01
Mature leaf width	−1.048^***^	24.5	**1.357** ^***^	**49.0**	−0.696^***^	23.4	0.969	*F*_3,8_ = 82.16	<0.0001
(B) Individual-based model (*N* = 213)									
Early leaf length	**−1.097** ^***^	**16.2**	0.709^***^	7.8	**−1.355** ^***^	**20.2**	0.457	*F*_4,196_ = 41.22	<0.0001
Mature leaf length	**−1.408** ^***^	**11.2**	0.537^***^	7.2	**−1.960** ^***^	**17.0**	0.425	*F*_6,197_ = 24.25	<0.0001
Early leaf width	−0.431^***^	6.1	**0.556** ^***^	**24.6**	ns	ns	0.314	*F*_3,197_ = 30.05	<0.0001
Mature leaf width	−0.423^***^	5.8	**0.498** ^***^	**22.4**	ns	ns	0.282	*F*_2,201_ = 39.44	<0.0001

*Standardized coefficients estimated for the macroclimatic variables are shown. PV1, percentage of variation explained by temperature seasonality; PV2, percentage of variation explained by annual precipitation; and PV3, percentage of variation explained by isothermality. ns, not significant, p > 0.10;*

** *p < 0.01;*

*** *p < 0.001.*

a *For early leaf width, the macroclimatic variable is annual mean temperature*.

The individual-based model yielded largely congruent results with moderate predictive power (Multiple *R*^2^ = 0.282–0.457, *p* < 0.0001 in all the cases; Table 2B). For early/mature leaf length, the three variables explained most of the variation were the same as those in population-based model; while for early/mature leaf width, two variables, temperature seasonality and annual precipitation, were significant predictors (Supplementary Table 9; Figures 6B1–B3). We also did the multiple regression using aridity index instead of annual precipitation, and the results were largely consistent (Supplementary Tables 10 and 11). Among the predictive bioclimatic variables, annual precipitation gave the highest correlation with longitude (Spearman’s rank test, *r* = 0.99, *N* = 12, *p* < 0.0001; Figure 6C2), followed by isothermality (*r* = 0.71, *p* = 0.012; Figure 6C3), but no correlation between temperature seasonality/annual mean temperature and longitude was detected (*p* = 0.30/0.13; Figure 6C1).

## Discussion

The adaptive function of leaf shape has been long debated, and here, we contribute empirical evidence to this debate. Our comprehensive analyses of leaf-shape variation, neutral genetic structure, micro- and macrohabitat in *O. diversifolia*, and two close relatives revealed three major findings. First, we found significant clinal variation of leaf shape in *O. diversifolia* across its distribution range in Nei Mongol, China. The three phenotypes also differ in leaflet-blade size, with phenotypes with more leaflets having smaller leaflet blades. Second, compared with *O. neimonggolica* and *O. leptophylla*, the genetic differentiation of populations in *O. diversifolia* at putatively neutral markers is relatively low, and there seems to be a lack of population genetic structure. Third, the clinal pattern of leaf-shape variation in *O. diversifolia* could be largely explained by three macroclimatic variables, temperature seasonality, annual precipitation, and isothermality. The three phenotypes of *O. diversifolia* also differ slightly in one microhabitat variable, the slope. These results indicate that the spatial pattern of leaf-shape variation in *O. diversifolia* is likely to result from natural selection driven by abiotic environmental factors. We discuss these results below and also used our results to tentatively explain the spatial patterns of the three species.

### Natural Selection and the Leaf-Shape Cline in *O. diversifolia*

We first revealed significant spatial variation of phenotypes in *O. diversifolia* populations: The proportion of 1 leaflet increases from west to east, while the proportions of 1–3 leaflets and 3 leaflets exhibit the reverse trend. However, the genetic differentiation at presumed neutral markers did not exhibit the same pattern. Compared with *O. neimonggolica* and *O. leptophylla*, analyses of variation within *O. diversifolia* showed a relatively high level of genetic diversity but with low levels of differentiation between populations (excluding population BT). Furthermore, despite the 252 km span of the 11 populations (comparable with the 255 km span of *O. leptophylla*, but larger than the 159 km span of *O. neimonggolica*; Figure 4C), we did not find a significant correlation between genetic distance and geographic distance, and we also failed to detect a hierarchical population structure (but note that both were revealed in *O. neimonggolica* with reduced geographical distance). These results indicate that there might be extensive gene flow among populations, even though some of them are geographically distinct and dominated by different leaf-shape phenotypes. The lack of clear differentiation and evidence of gene flow can prevent the evolution of clines, yet clines still evolved. This finding implies that spatially varying natural selection might contribute to the geographical cline of leaf shape despite the homogenizing effects of gene flow. Similar results were revealed in leaf-shape polymorphic *I. hederacea* (Campitelli and Stinchcombe, 2013), for which they attributed the phenotypic cline to natural selection in the face of gene flow as well.

The 1-leaflet-dominant population BT seems to be an exception in *O. diversifolia*. We revealed that it exhibits a low level of genetic diversity at both markers despite having a sample size comparable with that of other 1-leaflet-dominant populations (GXB and DT; Supplementary Tables 4 and 7). In addition, the microsatellite dataset showed that it exhibits a high level of genetic differentiation from the other populations (Figure 4D). These results indicate that gene flow between BT and the other populations is restricted, and this population may be heavily influenced by genetic drift, which might confound the effect of natural selection to purify leaf-shape variation. Indeed, our environmental association analysis revealed that the abiotic environment in this population is suitable for the 1-leaflet phenotype (Figure 6). From the cpDNA dataset, we found that the dominant haplotypes in BT are common in other populations of *O. diversifolia* (H15 and H17; Supplementary Figure 2). Consequently, BT might have experienced a founder event and could be an expanding edge in the east. In contrast, the other two populations dominated by 1-leaflet phenotype, GXB and DT, do not lack genetic diversity, and the microsatellite dataset even showed a decrease in expected heterozygosity westward (Figure 4A; and so do the population size; Figure 4B). The cpDNA dataset revealed a significantly negative estimate of Tajima’s *D* in *O. diversifolia*, which is associated with an accumulation of rare variants and hence are often interpreted as indicating rapid population expansion. The “star-like” pattern of haplotype network is also indicative for such event (Figure 3B; reviewed in Allcock and Strugnell, 2012). We thus expect a reverse westward colonization to harsher environments for the remaining 11 populations (dry *Stipa* grasslands toward semi-desert regions), and natural selection could play a role during this process.

### Potential Selective Agents Acting on Leaf Shape of *O. diversifolia*

Our environmental association analysis provided some cues about potential selective agents on leaf shape in *O. diversifolia*. We found that the clinal variation in leaf shape could be tentatively explained by three geographically varying macroclimatic variables: temperature seasonality, annual precipitation, and isothermality. In general, 1-leaflet plants with relatively larger leaflet-blade size predominate in regions with higher annual precipitation but lower temperature seasonality and isothermality, while 1–3 leaflet and 3-leaflet plants with reduced leaflet-blade size are more frequent in drier regions with pronounced temperature seasonality and isothermality. These results fit the ecophysiological predictions well. Concerning the thermoregulation theory, boundary layer thickness increases with effective leaf size, specifically mean length of a leaf in the direction of the wind (Givnish, 1979; Nobel, 2005). Lineage-specific genetic or developmental constraints may make reductions in effective leaf size *via* lobed or compound leaves more likely in some lineages, and *via* simple reductions in entire leaf size in others. Here, in *O. diversifolia*, leaf dissection did result in smaller leaf divisions, especially leaf length is reduced (Figures 2A,B), and thus, thinner boundary layer is possible, which can prevent the leaf from readily overheating, maintain warmer leaves in cold conditions, and enable plant survival in places with greater temperature change. Meanwhile, dissected leaves can also have advantages in dry environments if leaf width is reduced, *via* better water supply and lower hydraulic resistance (Givnish, 1979; Nicotra et al., 2011). In our study, plants with more leaflets did have reduced leaf width (Figures 2C,D), and much of the variance among populations could be explained by annual precipitation (Table 2; Figures 6A2,B2).

Our results are consistent with two previous studies using reciprocal transplant experiments at a reduced geographical scale to test for the adaptive feature of leaf shape (Ferris and Willis, 2018; Richards et al., 2019), both of which found dissected leaves performed better in dry environments. However, unlike the consistent adaptation to drought, previous studies found that lobed/dissected leaves are likely adaptive in either particularly hot or cold habitats. For example, lobed/dissected leaves were frequently found in the warmer low-altitude regions (*Achillea millefolium*, Gurevitch, 1988; the *Senecio*-hybrid zone, Brennan et al., 2009); meanwhile, analysis of latitudinal leaf-shape cline revealed increased lobing/dissection in northern cooler environments (*I. hederacea*, Campitelli and Stinchcombe, 2013; *Acer ginnala*, Zhu et al., 2015). Our result is thus the first to highlight the potential importance of temperature variability instead of mean temperature in the evolution of complex leaves. Further reciprocal transplant experiment would be helpful to directly test the fitness consequences of those different macroclimatic environments.

### Species Status and the Spatial Pattern of the Three *Oxytropis* Species

From our interspecies analyses, we revealed that the three *Oxytropis* species had significantly distinct leaf morphologies and exhibit substantial neutral genetic differentiation from one another (interspecific differentiation > intraspecific differentiation). These results indicate that these taxa are independently evolving units and should be recognized as separate, good species. This conclusion is particularly important for the species pair *O. neimonggolica* and *O. diversifolia*. The morphology of *O. neimonggolica* is much like the 1-leaflet phenotype of *O. diversifolia* (Figures 1A,D). *O. neimonggolica* was characterized by the 1-leaflet feature when first recognized as a new species (Chang and Zhao, 1981), while *O. diversifolia* was characterized by 3 leaflets and the phenotypes of 1 leaflet, 1–3 leaflets were not reported (see Flora of China, Zhu et al., 2010). Our genetic results confirmed the species status of these two, but the phenotypic description of *O. diversifolia* must be formally revised in the future.

Because the distributional patterns of *O. diversifolia* and *O. neimonggolica* are different from that of *O. diversifolia* and *O. leptophylla* (allopatric vs. parapatric), we tentatively provide different explanations. For the allopatric pair of *O. neimonggolica* and *O. diversifolia*, the Hetao Plain (the region from Bayannuur to Baotou with relatively low altitude; Figure 1G) seems to be a natural gap separating them. The Hetao Plain is a fertile floodplain with good irrigation, and it is intensively farmed. Consequently, the Hetao Plain might act as a barrier which could impede gene flow and facilitate genetic divergence between the two species. We noticed that, although 1-leaflet phenotype is nearly fixed in all populations of *O. neimonggolica*, population divergence at both markers still exists (Supplementary Table 5; Figure 5; probably due to the west–east geographic separation in Helan Mountains), indicating stabilizing selection may maintain the 1-leaflet phenotype in this species. The parapatric *O. diversifolia* and *O. leptophylla* only meet close to the longitude of 110°E (Figure 1G), where hybridization between them occurred. The morphological and genetic analyses of hybrid swarms indicate a potential outcome of introgression between species (Wang et al., unpubl. Res.), and it would be interesting to test whether leaf-shape polymorphism in *O. diversifolia* was obtained through introgression from *O. leptophylla*. It was suggested that adaptive divergence could make the hybrid zone typically a tension zone, and the transition from one lineage to another could show a narrow cline (Barton and Hewitt, 1985; Barton and Gale, 1993). We therefore suspect that there might be strong natural selection maintaining the sharp transition between the two species.

Although we can tentatively explain the spatial pattern of these three species, it is still confusing that the general trend of leaf-shape variation at the interspecies level is opposite to that observed at the intraspecies level. With decreasing water availability from east to west, leaflet production increases within *O. diversifolia* while decreasing among species. We suspect there might be some uninvestigated factors (e.g., soil chemistry) driving interspecific patterns of leaf-shape variation. Polyploidy may also be considered to explain leaf-shape variation at intra- and interspecific level. For example, three main cytotypes (di-, tetra-, and hexaploids) of *Senecio carniolicus* (Asteraceae) and the diploid *S. incanus* differ in leaf dissection (Suda et al., 2007; Flatscher et al., 2015). But the information of ploidy-level variation is currently not available in our system. Furthermore, because the evolution of leaf shape is a function of both phylogenetic history and adaptation to contemporary environmental conditions (Givnish, 1987; Nicotra et al., 2011), interspecific leaf-shape evolution needs to be further investigated through more detailed phylogeographic analyses and placed in a phylogenetic context. The genus *Oxytropis* is well known for recent rapid radiation in Asian mountains (Kholina et al., 2016; Shahi Shavvon et al., 2017), producing a high-resolution phylogeny and exploring the role of natural selection during evolutionary radiation in this genus remains a challenge.

## Conclusion

The intraspecific leaf-shape variation in *O. diversifolia* appears to follow an adaptive longitudinal cline, and the spatially varying bioclimatic characteristics (i.e., temperature seasonality, annual precipitation, and isothermality) seem to be important producing the cline. Future experimental work evaluating leaf ecophysiological function is required to confirm the proposed agents of selection. This can be achieved by measuring and testing whether three leaf-shape phenotypes differ in leaf functional traits related to water physiological characteristics (e.g., leaf thickness, vein density, and C-isotope composition as a measure of intrinsic water-use efficiency; from Pérez-Harguindeguy et al., 2013), and whether potential leaf thermoregulation differs by measuring leaf temperature (e.g., Campitelli et al., 2013). Moreover, we suggest that the interspecific pattern of leaf-shape variation may be explained by both local adaptation and historical events. Producing a robust phylogeny of the genus *Oxytropis* using whole-plastome sequence data, and investigating the situations in which leaf-shape diversification arises may provide useful insights into leaf-shape evolution in this system.

## Data Availability Statement

The datasets presented in this study can be found in online repositories. The names of the repository/repositories and accession number(s) can be found at https://www.ncbi.nlm.nih.gov/genbank/, MN950995-MN952199.

## Author Contributions

HW and Z-YC designed the study. HW collected the samples in the field, analyzed the data, and wrote the manuscript. P-LL was responsible for the field work, collected the voucher specimens, and participated in the cpDNA data analyses. JL and HY measured the leaf-morphological traits and conducted the cpDNA sequencing and microsatellite genotyping under the supervision of HW and Z-YC. QL contributed to the original idea and analysis of micro- and macrohabitat data. All authors approved the final version of the manuscript.

## Conflict of Interest

The authors declare that the research was conducted in the absence of any commercial or financial relationships that could be construed as a potential conflict of interest.

## Publisher’s Note

All claims expressed in this article are solely those of the authors and do not necessarily represent those of their affiliated organizations, or those of the publisher, the editors and the reviewers. Any product that may be evaluated in this article, or claim that may be made by its manufacturer, is not guaranteed or endorsed by the publisher.

## Footnotes

^1^ http://www.geneious.com

^2^ http://tree.bio.ed.ac.uk/software/figtree/

^3^ http://www.worldclim.org
