# Supplementary material for: Why More Leaflets? The Role of Natural Selection in Shaping the Spatial Pattern of Leaf-Shape Variation in Oxytropis diversifolia (Fabaceae) and Two Close Relatives
Source: Front Plant Sci. 2021 Aug 19;12:681962. doi: 10.3389/fpls.2021.681962 (PMC8416669; doi:10.3389/fpls.2021.681962)
Supplement: Supplementary file 1 [file Data_Sheet_1.docx]

Supplementary Material

# Supplementary Figures


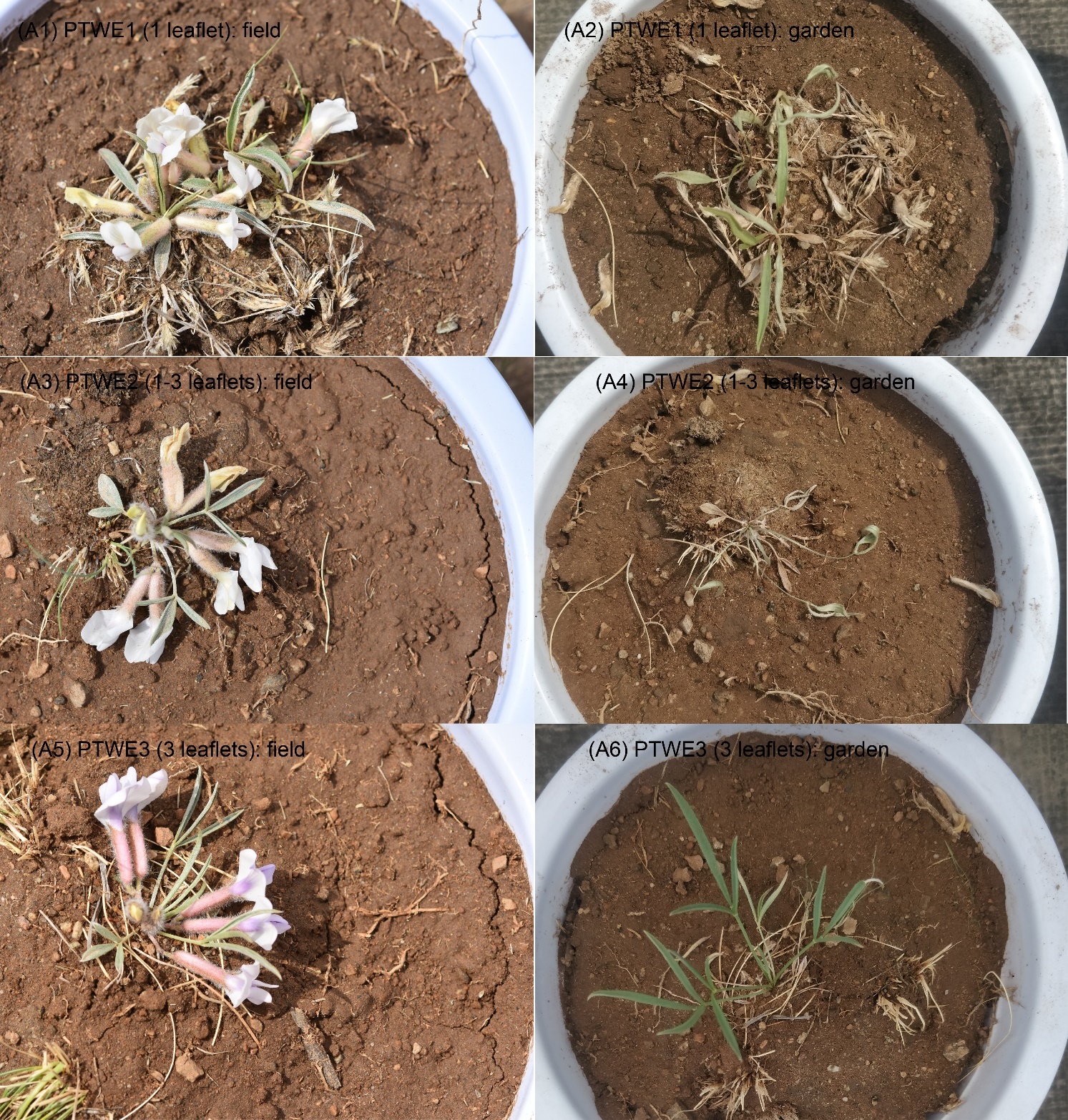


**Supplementary Figure 1****A.** Common Garden experiment showing leaf morphology of field-transplanted individuals from population PTWE. **(A1, A3, A5)** morphology in the field; **(A2, A4, A6)** morphology in the common garden one month after transplanting. Plants were growing in soil of the original site.

**
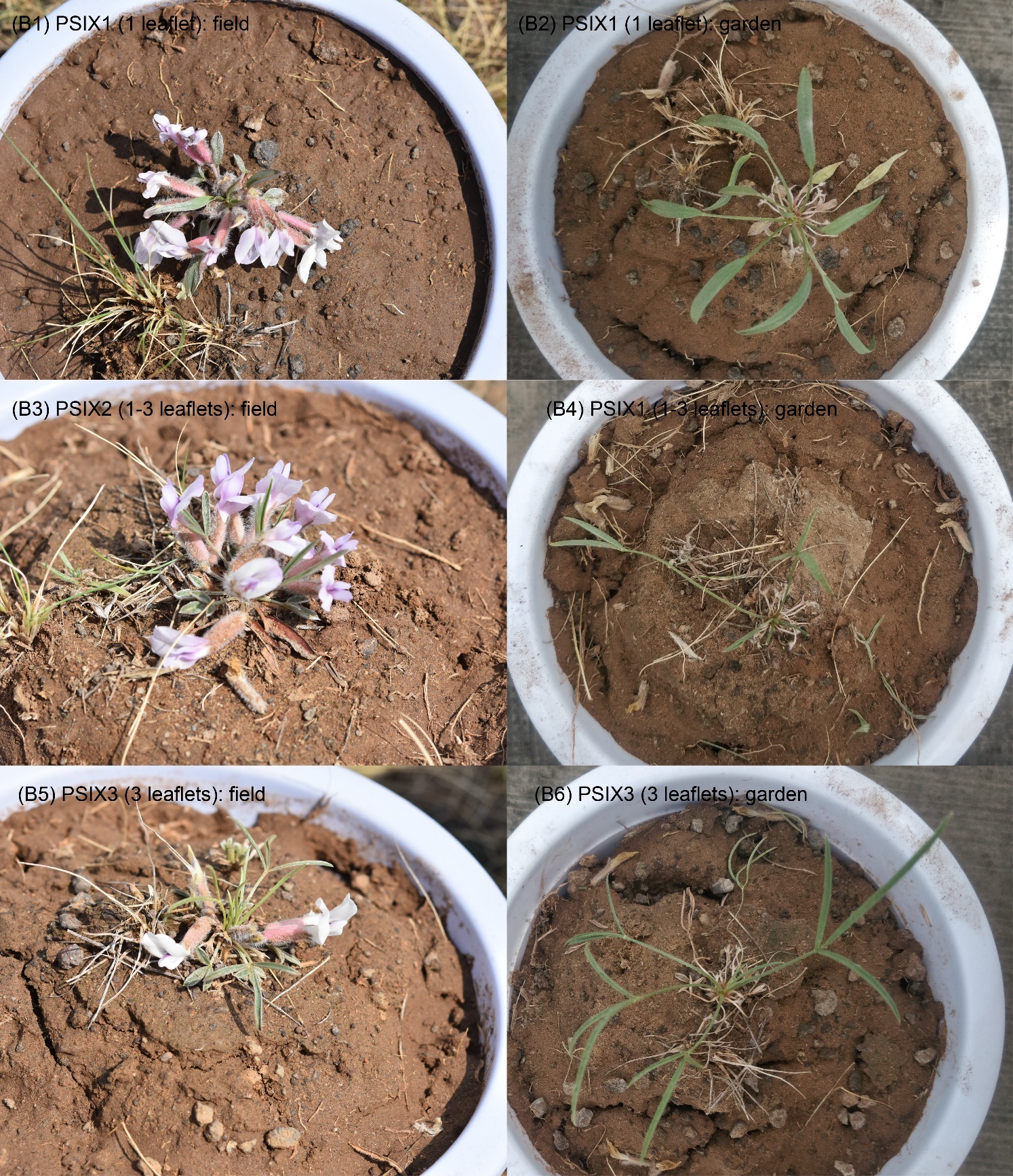
**

**Supplementary Figure 1B.** Common Garden experiment showing leaf morphology of field-transplanted individuals from population PSIX. **(B1, B3, B5)** morphology in the field; **(B2, B4, B6)** morphology in the common garden one month after transplanting. Plants were growing in soil of the original site.

**
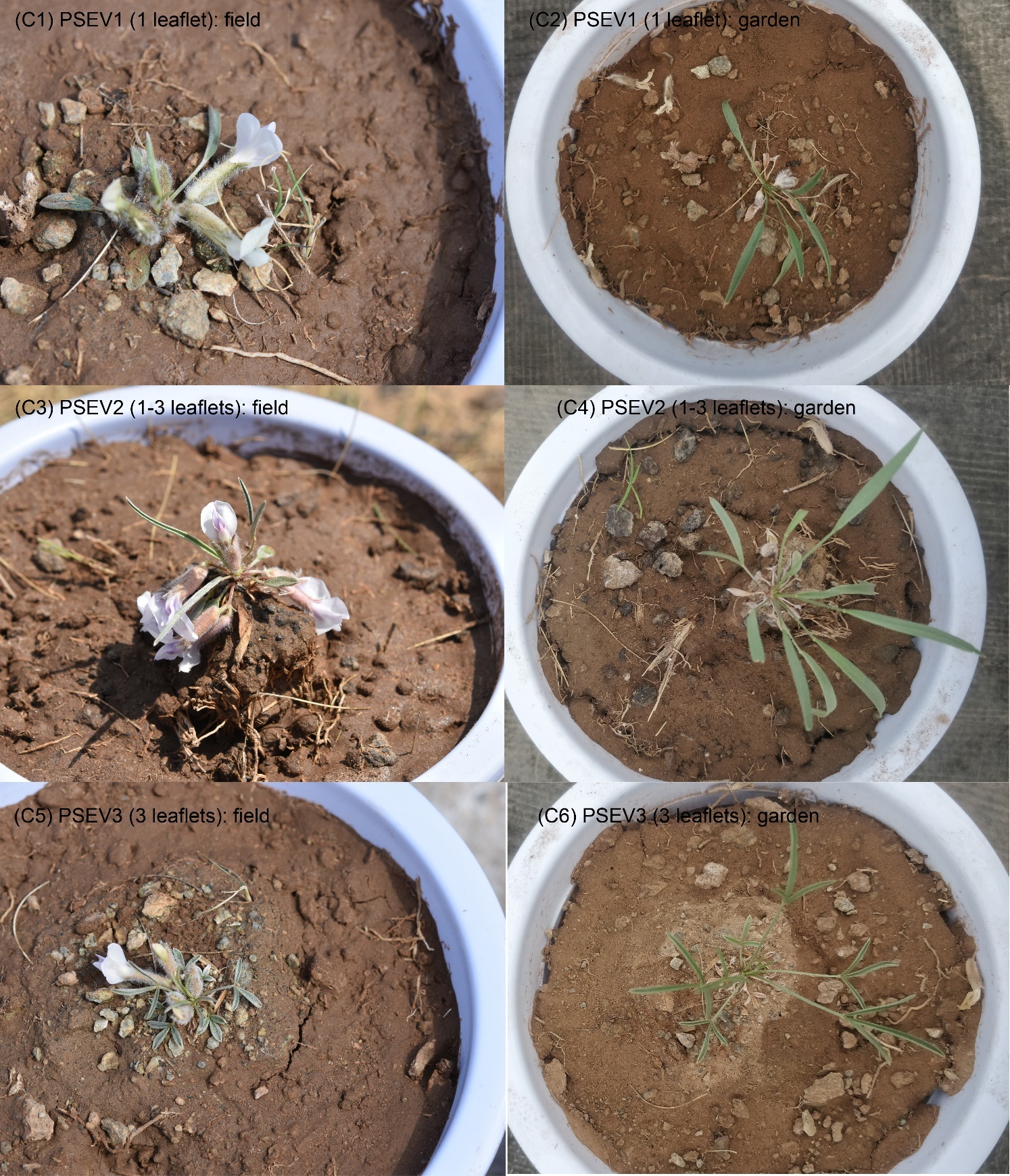
**

**Supplementary Figure 1C.** Common Garden experiment showing leaf morphology of field-transplanted individuals from population PSEV. **(C1, C3, C5)** morphology in the field; **(C2, C4, C6)** morphology in the common garden one month after transplanting. Plants were growing in soil of the original site.

**
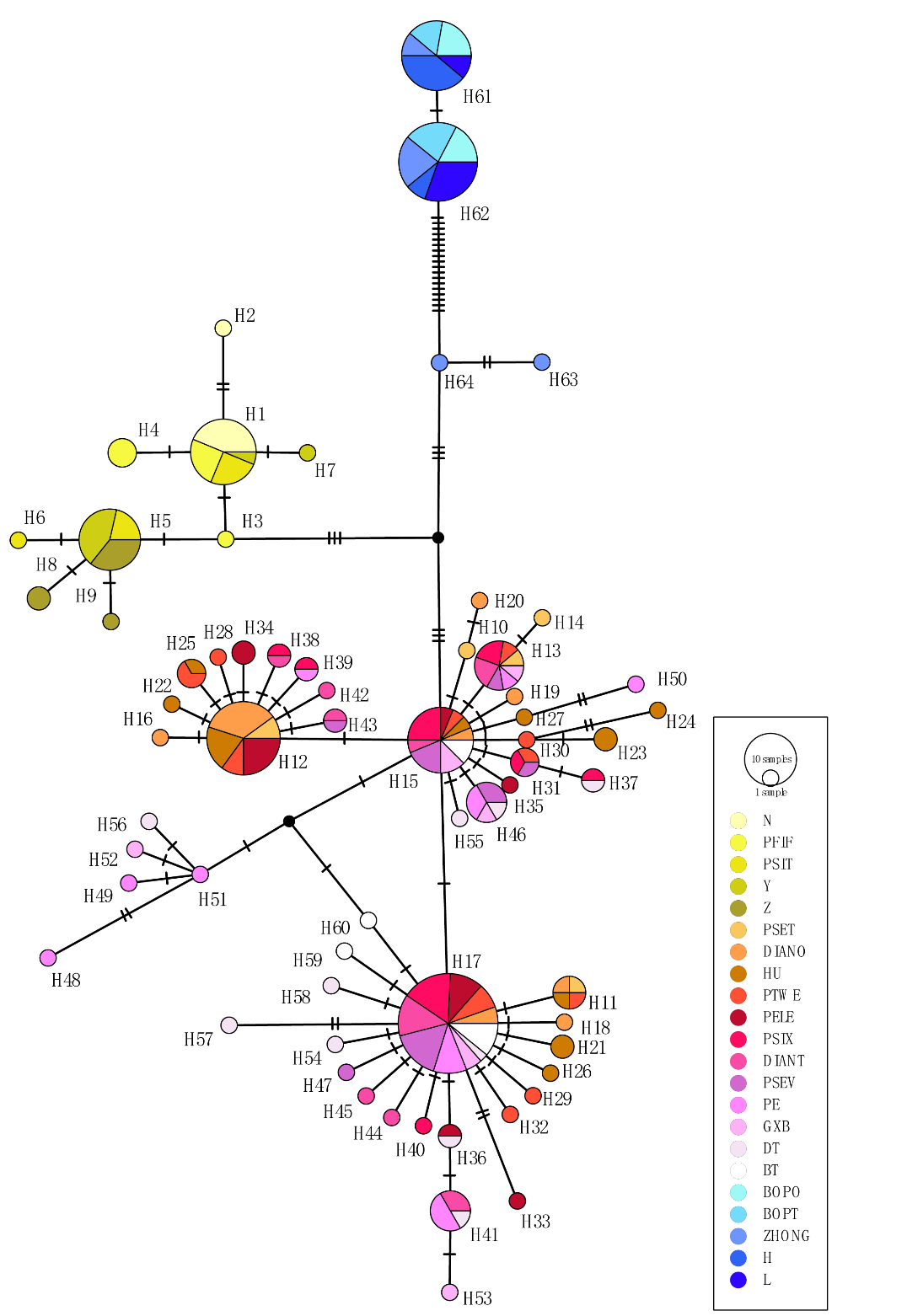
**

**Supplementary Figure 2.** Network-derived genealogical relationships of the 64 cpDNA haplotypes. This figure is the same as Figure 3B but with colors representing different populations. Populations N, PFIF, PSIT, Y, and Z are *O. neimonggolica*; populations PSET, DIANO, HU, PTWE, PELE, PSIX, DIANT, PSEV, PE, GXB, DT, and BT are *O. diversifolia*; populations BOPO, BOPT, ZHONG, H, and L are *O. leptophylla*.


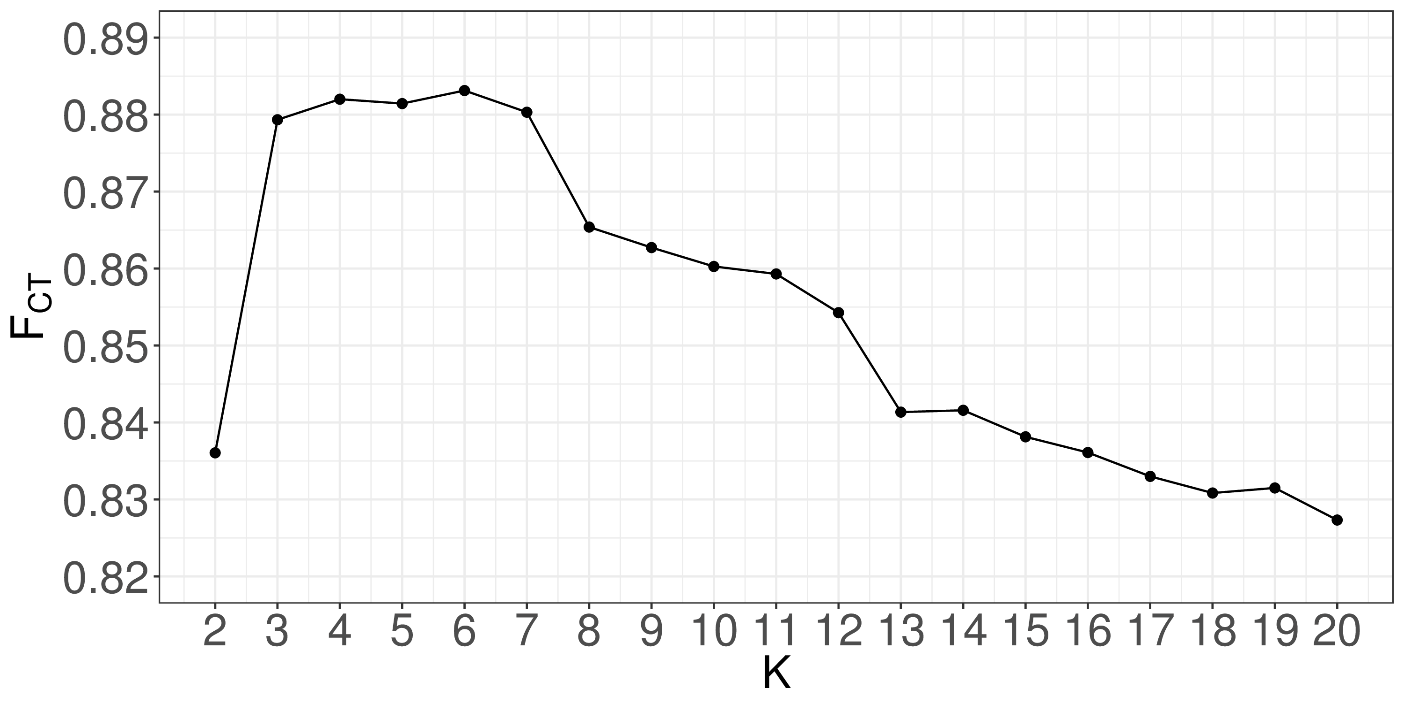


**Supplementary Figure 3.** Inference of the number of clusters (*K*), from SAMOVA performed on cpDNA dataset for all 22 populations combined. The optimum number of clusters (the *K* that best fit the data) was estimated by calculating *F*_CT_ value given each *K* (10 replicates) (Dupanloup et al., 2002). The best *K* inferred was 3 when *F*_CT_ reached a plateau.


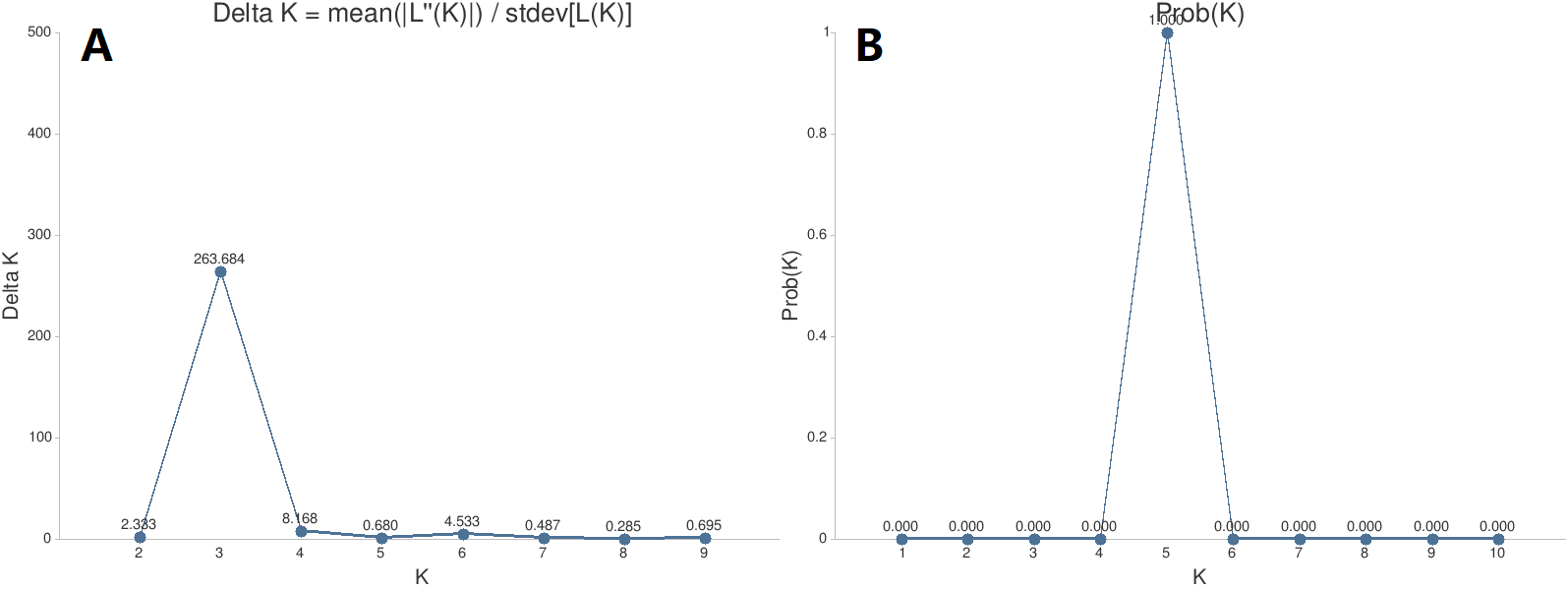


**Supplementary Figure 4.** Bayesian inference of the number of clusters (*K*), from STRUCTURE analysis of nuclear microsatellite dataset for all 22 populations combined. The optimum number of clusters (the *K* that best fit the data) was estimated using **(A)** the distribution of delta *K* based on L(*K*) (Evanno et al., 2005) and **(B)** the posterior probability of the data given each *K* (10 replicates) (Pritchard et al., 2000).


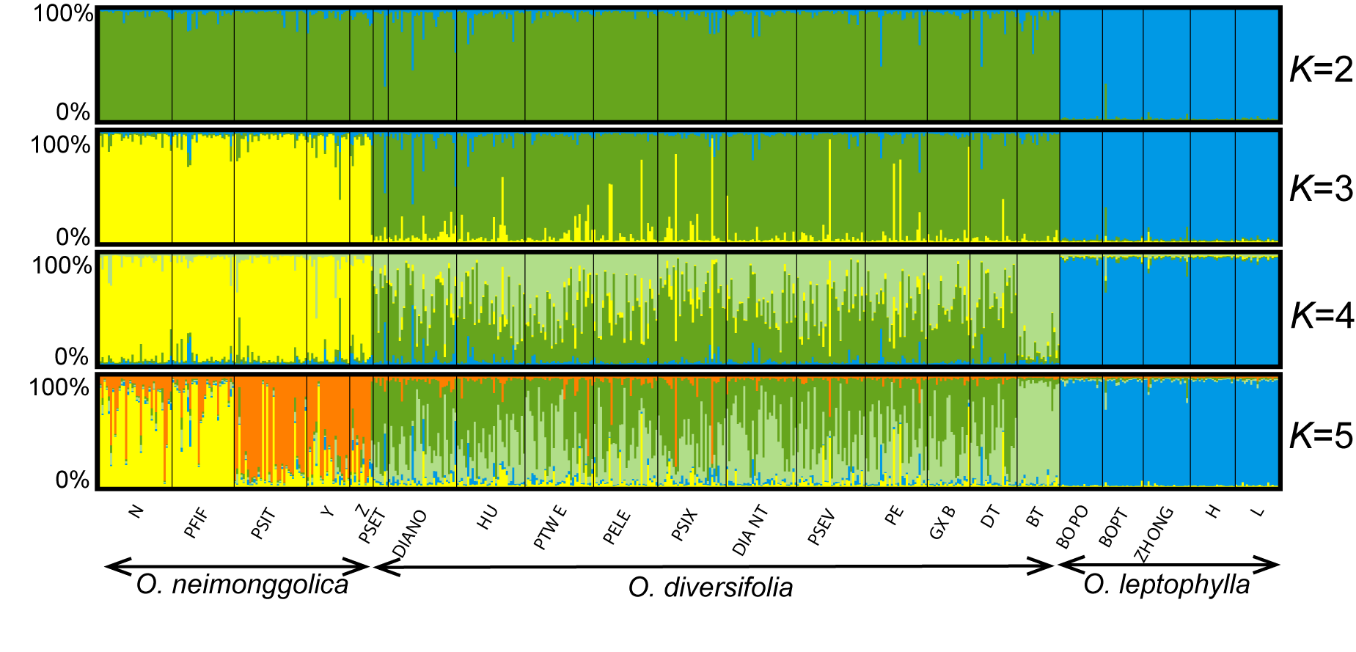


**Supplementary Figure 5.** InStruct results for 22 *Oxytropis* populations based on nuclear microsatellite dataset (*K* = 2 – 5). The small black lines separate populations, named at the bottom of the graph. Each individual is a small bar with color coded according to probability of clustering in a particular group. Populations are ordered west to east within each species.

# Supplementary Tables

**Supplementary Table 1.** Characteristics of the 22 *Oxytropis* populations sampled. Populations are sorted by increasing longitude (from west to east in Figure 1G) for each species, respectively.

| **No.** | **Species** | **Pop.** | **Collection locality** | **Voucher no.^a^** | **Year** | **GPS** | | **Alt.**  **(m)** | **Size^b^** | **Phenotype proportion^c^** | | | | |
| --- | --- | --- | --- | --- | --- | --- | --- | --- | --- | --- | --- | --- | --- | --- |
|  |  |  |  |  |  | **Lat.**  **(°N)** | **Long.**  **(°E)** |  |  | **1 leaflet** | **1-3 leaflets** | **3 leaflets** | **3-5 leaflets** | **5-13 leaflets** |
| 1 | *O. neimonggolica* | N | Alxa Zuoqi, Nei Mongol | Chang2016051 | 2016 | 38.6603 | 105.8042 | 1964 | 122 | 100% (122) | 0 | 0 | 0 | 0 |
| 2 | *O. neimonggolica* | PFIF | Alxa Zuoqi, Nei Mongol | Chang2016056 | 2016 | 38.8534 | 105.8554 | 2100 | 142 | 99.3% (141) | 0.7% (1) | 0 | 0 | 0 |
| 3 | *O. neimonggolica* | PSIT | Alxa Zuoqi, Nei Mongol | Chang2016059 | 2016 | 39.0388 | 105.9820 | 1866 | 153 | 100% (153) | 0 | 0 | 0 | 0 |
| 4 | *O. neimonggolica* | Y | Shizuishan City, Ningxia | Chang2017005 | 2017 | 39.0316 | 106.1209 | 1830 | 57 | 100% (57) | 0 | 0 | 0 | 0 |
| 5 | *O. neimonggolica* | Z | Wuhai City, Nei Mongol | Chang2017017 | 2017 | 39.8058 | 106.9163 | 1687 | 12 | 100% (12) | 0 | 0 | 0 | 0 |
| 6 | *O. diversifolia* | PSET | Urad Houqi, Nei Mongol | Chang2016076 | 2016 | 41.4775 | 106.9509 | 1604 | 9 | 44.4% (4) | 22.2% (2) | 33.3% (3) | 0 | 0 |
| 7 | *O. diversifolia* | DIANO | Urad Zhongqi, Nei Mongol | Chang2017030 | 2017 | 41.5207 | 107.6290 | 1480 | 76 | 5.3% (4) | 26.3% (20) | 68.4% (52) | 0 | 0 |
| 8 | *O. diversifolia* | HU | Urad Zhongqi, Nei Mongol | Chang2017034 | 2017 | 41.5694 | 108.3052 | 1319 | 57 | 8.8% (5) | 63.1% (36) | 28.1% (16) | 0 | 0 |
| 9 | *O. diversifolia* | PTWE | Urad Zhongqi, Nei Mongol | Chang2016029 | 2017 | 41.6102 | 108.4500 | 1441 | 36 | 25.0% (9) | 33.3% (12) | 41.7% (15) | 0 | 0 |
| 10 | *O. diversifolia* | PELE | Urad Zhongqi, Nei Mongol | Chang2016027 | 2017 | 41.5265 | 108.6436 | 1299 | 45 | 35.5% (16) | 37.8% (17) | 26.7% (12) | 0 | 0 |
| 11 | *O. diversifolia* | PSIX | Urad Zhongqi, Nei Mongol | Chang2016022 | 2017 | 41.4911 | 108.9556 | 1352 | 170 | 15.9% (27) | 18.2% (31) | 65.9% (112) | 0 | 0 |
| 12 | *O. diversifolia* | DIANT | Urad Zhongqi, Nei Mongol | Chang2016020 | 2017 | 41.4304 | 109.1337 | 1453 | 121 | 20.7% (25) | 9.1% (11) | 70.2% (85) | 0 | 0 |
| 13 | *O. diversifolia* | PSEV | Urad Zhongqi, Nei Mongol | Chang2016023 | 2017 | 41.4224 | 109.2560 | 1493 | 98 | 23.5% (23) | 35.7% (35) | 40.8% (40) | 0 | 0 |
| 14 | *O. diversifolia* | PE | Urad Zhongqi, Nei Mongol | Chang2016024 | 2017 | 41.4011 | 109.3644 | 1546 | 249 | 48.2% (120) | 37.7% (94) | 14.1% (35) | 0 | 0 |
| 15 | *O. diversifolia* | GXB | Guyang County, Nei Mongol | Chang2016018 | 2017 | 41.2838 | 109.8094 | 1560 | 82 | 95.1% (78) | 4.9% (4) | 0 | 0 | 0 |
| 16 | *O. diversifolia* | DT | Damao Qi, Nei Mongol | Chang2016015 | 2017 | 41.4311 | 109.9689 | 1554 | 318 | 97.5% (310) | 2.5% (8) | 0 | 0 | 0 |
| 17 | *O. diversifolia* | BT | Baotou City, Nei Mongol | Chang2016005 | 2017 | 40.7159 | 110.1027 | 1338 | 125 | 97.6% (122) | 2.4% (3) | 0 | 0 | 0 |
| 18 | *O. leptophylla* | BOPO | Guyang County, Nei Mongol | Chang2016011 | 2017 | 41.0899 | 110.0500 | 1418 | 33 | 3.0% (1) | 0 | 3.0% (1) | 9.1% (3) | 84.9% (28) |
| 19 | *O. leptophylla* | BOPT | Guyang County, Nei Mongol | Chang2016088 | 2017 | 41.0693 | 110.1042 | 1422 | 42 | 0 | 0 | 0 | 0 | 100%  (42) |
| 20 | *O. leptophylla* | ZHONG | Wuchuan County, Nei Mongol | Chang2018090 | 2018 | 40.9567 | 111.5236 | 1554 | 53 | 0 | 0 | 0 | 0 | 100%  (53) |
| 21 | *O. leptophylla* | H | Siziwang Qi, Nei Mongol | Chang2018105 | 2018 | 41.8767 | 111.9617 | 1500 | 25 | 0 | 0 | 0 | 4.0% (1) | 96.0% (24) |
| 22 | *O. leptophylla* | L | Qahar Youyi Qianqi, Nei Mongol | Chang2018120 | 2018 | 41.0958 | 113.0917 | 1400 | 26 | 0 | 0 | 0 | 0 | 100% (26) |

^a^ All voucher specimens are deposited in the Northwest A&F University Herbarium (WUK), Yangling, Shaanxi, China.

^b^ Size: estimated population size, i.e., the total number of individuals in the population.

^c^ Phenotype proportion: the proportion of each phenotype in the population, with the number of individuals for each phenotype in brackets.

**Supplementary Table 2.** Inter- and intraspecific summary statistics for leaf-morphological traits measured from 22 *Oxytropis* populations

|  | **Interspecies** | | | | |  | **Intraspecies (*O. diversifolia*)** | | | | |
| --- | --- | --- | --- | --- | --- | --- | --- | --- | --- | --- | --- |
|  | ***O.***  ***neimonggolica***  ***N* = 128**  **(1 leaflet)** | ***O.***  ***diversifolia***  ***N* = 313**  **(****1, 1-3, 3 leaflets)** | ***O.***  ***leptophylla***  ***N* = 96**  **(5-13 leaflets)** | ***P*-value** | **Pop.** |  | **1 leaflet**  ***N* = 116** | **1-3 leaflets**  ***N* = 99** | **3 leaflets**  ***N* = 98** | ***P*-value** | **Pop.** |
| Early leaf length | 10.5 (4.15) a | 6.85 (1.75) b | 4.89 (1.58) b | <0.001 | 57.5% |  | 7.72 (2.03) a´ | 6.58 (1.38) b´ | 6.12  (1.25) b´ | <0.0001 | 40.7% |
| Mature leaf length | 18.4 (5.58) a | 13.5 (3.57) b | 12.3 (3.98) c | <0.05 | 52.9% |  | 15.3 (3.94) a´ | 12.8 (3.25) b´ | 12.2  (2.39) b´ | <0.0001 | 40.0% |
| Early leaf width | 3.48 (1.12) a | 2.09 (0.618) b | 1.16 (0.236) c | <0.0001 | 39.5% |  | 2.50 (0.617) a´ | 1.98 (0.537) b´ | 1.73 (0.384) c´ | <0.0001 | 34.3% |
| Mature leaf width | 4.26 (1.17) a | 2.00 (0.554) b | 1.33 (0.311) c | <0.0001 | 25.9% |  | 2.32 (0.595) a´ | 1.90 (0.485) b´ | 1.74 (0.355) b´ | <0.0001 | 18.2% |

Data are presented as mean (s.d.); the four leaf-morphological traits are in mm. *N*, number of individuals measured. *P*-values indicate the significance of differences among species (interspecies) or among phenotypes (intraspecies) in linear mixed-effects models, and percentage variance explained by the random factor “population” is given. Different letters indicate significant differences of pairwise comparisons (*P* < 0.05, Tukey’s HSD test).

**Supplementary Table 3.** Primer sequences of cpDNA intergenic spacer regions amplified in this study. T_a_, annealing temperature.

| **Locus** | **Align. length (bp)** | **Primer sequence (5'-3')** | **T_a_ (°C)** | **Reference** |
| --- | --- | --- | --- | --- |
| *trn*T-*psb*D | 896 | F: CCCTTTTAACTCAGTGGTAG | 60 | Shaw *et al*. (2007) |
|  |  | R: CTCCGTARCCAGTCATCCATA |  |  |
| *pet*N-*psb*M | 863 | F: ATGGATATAGTAAGTCTYGCTTGGGC | 54 | Shaw *et al*. (2005) |
|  |  | R: ATGGAAGTAAATATTCTYGCATTTATTGCT |  |  |
| *trn*S-*trn*G | 519 | F: AACTCGTACAACGGATTAGCAATC | 53 | Shaw *et al*. (2007); |
|  |  | R: TTTTACCACTAAACTATACCCGC |  | Shaw *et al*. (2005) |
| *psb*E-*pet*L | 794 | F: TATCGAATACTGGTAATAATATCAGC | 50 | Shaw *et al*. (2007) |
|  |  | R: AGTAGAAAACCGAAATAACTAGTTA |  |  |
| *rpl*16 intron | 777 | F: GCTATGCTTAGTGTGTGACTCGTTG | 60 | Shaw *et al*. (2005) |
|  |  | R: CCCTTCATTCTTCCTCTATGTTG |  |  |
| **Total** | **3849** |  |  |  |

Shaw J, Lickey EB, Beck JT, et al. 2005. The tortoise and the hare II: relative utility of 21 noncoding chloroplast DNA sequences for phylogenetic analysis. *American Journal of Botany* **92**: 142–166. doi: 10.3732/ajb.92.1.142

Shaw J, Lickey EB, Schilling EE, Small RL. 2007. Comparison of whole chloroplast genome sequences to choose noncoding regions for phylogenetic studies in angiosperms: the tortoise and the hare III. *American Journal of Botany* **94**: 275–288. doi: 10.3732/ajb.94.3.275

**Supplementary Table 4.** Summary of nucleotide diversity for cpDNA dataset. *N*_1_, number of 1-leaflet individuals sequenced; *N*_2_, number of 1-3 leaflets individuals sequenced; *N*_3_, number of 3-leaflets individuals sequenced; *N*_4_, number of 3-5 leaflets individuals sequenced; *N*_5_, number of 5-13 leaflets individuals sequenced; *N*, total number of individuals sequenced. *S*, number of segregating sites; *h*, number of haplotypes; *h*_p_, number of private haplotypes (i.e., haplotypes that are fixed in only one population); *Hd*, haplotype diversity; *π*, nucleotide diversity. ns, not significant, *P* > 0.10; + *P* > 0.05; * *P* < 0.05; ** *P* < 0.01; *** *P*< 0.001.

| **Population** | ***N*_1_** | ***N*_2_** | ***N*_3_** | ***N*_4_** | ***N*_5_** | ***N*** | ***S*** | ***h*** | ***h*_p_** | ***Hd*** | ***π*** | **Tajima’s *D*** | **Fu and Li’s *D****** | **Fu and Li’s *F****** |
| --- | --- | --- | --- | --- | --- | --- | --- | --- | --- | --- | --- | --- | --- | --- |
| **(1) *O. neimonggolica*** | **40** | **0** | **0** | **0** | **0** | **40** | **9** | **9** | **7** | **0.724** | **0.00041** | **-0.838ns** | **-1.773ns** | **-1.733ns** |
| N | 8 | 0 | 0 | 0 | 0 | 8 | 2 | 2 | 1 | 0.250 | 0.00014 | -1.310ns | -1.410ns | -1.514ns |
| PFIF | 8 | 0 | 0 | 0 | 0 | 8 | 2 | 3 | 2 | 0.679 | 0.00022 | 0.069ns | -0.149ns | -0.108ns |
| PSIT | 8 | 0 | 0 | 0 | 0 | 8 | 3 | 3 | 1 | 0.679 | 0.00039 | 0.839ns | 0.301ns | 0.466ns |
| Y | 8 | 0 | 0 | 0 | 0 | 8 | 3 | 3 | 1 | 0.464 | 0.00031 | -0.177ns | 0.301ns | 0.209ns |
| Z | 8 | 0 | 0 | 0 | 0 | 8 | 2 | 3 | 2 | 0.607 | 0.00019 | -0.448ns | -0.149ns | -0.238ns |
| **(2) *O. diversifolia*** | **55** | **47** | **45** | **1** | **0** | **154** | **54** | **51** | **37** | **0.910** | **0.00063** | **-2.339**** | **-5.798**** | **-5.187**** |
| PSET | na | na | na | 0 | 0 | 6 | 6 | 5 | 2 | 0.933 | 0.00067 | -0.496ns | -0.416ns | -0.463ns |
| DIANO | 3 | 4 | 7 | 1 | 0 | 15 | 8 | 8 | 4 | 0.790 | 0.00049 | -1.060ns | -1.737ns | -1.781ns |
| HU | 4 | 6 | 5 | 0 | 0 | 15 | 12 | 10 | 6 | 0.924 | 0.00075 | -1.141ns | -1.282ns | -1.428ns |
| PTWE | 4 | 6 | 5 | 0 | 0 | 15 | 10 | 11 | 4 | 0.952 | 0.00061 | -1.119ns | -1.595ns | -1.681ns |
| PELE | 6 | 3 | 6 | 0 | 0 | 15 | 7 | 7 | 3 | 0.838 | 0.00051 | -0.542ns | -0.972ns | -0.981ns |
| PSIX | 4 | 6 | 7 | 0 | 0 | 17 | 8 | 8 | 1 | 0.838 | 0.00046 | -1.075ns | -0.799ns | -1.008ns |
| DIANT | 5 | 5 | 5 | 0 | 0 | 15 | 10 | 9 | 3 | 0.886 | 0.00063 | -1.001ns | -0.734ns | -0.925ns |
| PSEV | 5 | 5 | 5 | 0 | 0 | 15 | 7 | 7 | 1 | 0.819 | 0.00040 | -1.145ns | -1.220ns | -1.375ns |
| PE | 6 | 4 | 5 | 0 | 0 | 15 | 15 | 9 | 4 | 0.905 | 0.00093 | -1.172ns | -1.363ns | -1.506ns |
| GXB | 6 | 2 | 0 | 0 | 0 | 8 | 9 | 6 | 2 | 0.929 | 0.00071 | -1.375ns | -1.566ns | -1.686ns |
| DT | 6 | 4 | 0 | 0 | 0 | 10 | 14 | 10 | 5 | 1.000 | 0.00092 | -1.610+ | -1.878+ | -2.041+ |
| BT | 6 | 2 | 0 | 0 | 0 | 8 | 3 | 4 | 2 | 0.750 | 0.00026 | -0.525ns | -0.176ns | -0.282ns |
| **(3) *O. leptophylla*** | **0** | **1** | **0** | **4** | **38** | **43** | **21** | **4** | **2** | **0.550** | **0.00062** | **-1.779ns** | **1.008ns** | **0.074ns** |
| BOPO | 0 | 1 | 0 | 1 | 6 | 8 | 1 | 2 | 0 | 0.571 | 0.00016 | 1.444ns | 0.888ns | 1.100ns |
| BOPT | 0 | 0 | 0 | 1 | 7 | 8 | 1 | 2 | 0 | 0.571 | 0.00016 | 1.167ns | 0.888ns | 1.032ns |
| ZHONG | 0 | 0 | 0 | 0 | 9 | 9 | 21 | 4 | 2 | 0.694 | 0.00218 | 0.068ns | 1.210ns | 1.047ns |
| H | 0 | 0 | 0 | 2 | 7 | 9 | 1 | 2 | 0 | 0.389 | 0.00011 | 0.156ns | 0.840ns | 0.748ns |
| L | 0 | 0 | 0 | 0 | 9 | 9 | 1 | 2 | 0 | 0.389 | 0.00011 | 0.156ns | 0.840ns | 0.748ns |
| **Total** | **95** | **48** | **45** | **5** | **38** | **237** | **88** | **64** | **46** | **0.940** | **0.00286** | **-0.894ns** | **-4.564**** | **-3.450**** |

**Supplementary Table 5.** SAMOVA results from *K*=2 to *K*=7 based on cpDNA haplotypes. The optimal value for *K* was 3.

| **Species** | **Population** | ***K* = 2** | ***K* = 3** | ***K* = 4** | ***K* = 5** | ***K* = 6** | ***K* = 7** |
| --- | --- | --- | --- | --- | --- | --- | --- |
| *O. neimonggolica* | N | 1 | **1** | 1 | 1 | 1 | 1 |
|  | PFIF | 1 | **1** | 1 | 1 | 1 | 1 |
|  | PSIT | 1 | **1** | 2 | 2 | 2 | 2 |
|  | Y | 1 | **1** | 2 | 2 | 2 | 2 |
|  | Z | 1 | **1** | 2 | 3 | 3 | 3 |
| *O. diversifolia* | PSET | 1 | **2** | 3 | 4 | 4 | 4 |
|  | DIANO | 1 | **2** | 3 | 4 | 4 | 4 |
|  | HU | 1 | **2** | 3 | 4 | 4 | 4 |
|  | PTWE | 1 | **2** | 3 | 4 | 4 | 4 |
|  | PELE | 1 | **2** | 3 | 4 | 4 | 4 |
|  | PSIX | 1 | **2** | 3 | 4 | 4 | 4 |
|  | DIANT | 1 | **2** | 3 | 4 | 4 | 4 |
|  | PSEV | 1 | **2** | 3 | 4 | 4 | 4 |
|  | PE | 1 | **2** | 3 | 4 | 4 | 4 |
|  | GXB | 1 | **2** | 3 | 4 | 4 | 4 |
|  | DT | 1 | **2** | 3 | 4 | 4 | 4 |
|  | BT | 1 | **2** | 3 | 4 | 4 | 4 |
| *O. leptophylla* | BOPO | 2 | **3** | 4 | 5 | 5 | 5 |
|  | BOPT | 2 | **3** | 4 | 5 | 5 | 5 |
|  | ZHONG | 2 | **3** | 4 | 5 | 6 | 6 |
|  | H | 2 | **3** | 4 | 5 | 5 | 7 |
|  | L | 2 | **3** | 4 | 5 | 5 | 7 |

**Supplementary Table 6.** Microsatellite loci information. All of the loci were retrieved from Wang et al. (2018). Annealing temperature was 56℃ for all loci. *N*, total number of individuals with successful amplification; *A*, total number of alleles; *N*_a_, number of alleles per population; *H*_o_, observed heterozygosity; *H*_e_, expected heterozygosity; *F*_IS_, fixation index; *F*_ST_, population differentiation. For those genetic diversity parameters, data are presented as mean value averaged across all 22 populations.

| **No.** | **Locus** | **Repeat motif** | **Allele size range (bp)** | **Dye**  **(group)** | ***N*** | ***A*** | ***N*_a_** | ***H*_o_** | ***H*_e_** | ***F*_IS_** | ***F*_ST_** |
| --- | --- | --- | --- | --- | --- | --- | --- | --- | --- | --- | --- |
| 1 | N745892 | (ATAG)_12_ | 148–282 | 6-FAM (1) | 469 | 47 | 12.2 | 0.438 | 0.853 | 0.461 | 0.041 |
| 2 | N145635 | (GAG)_12_ | 87–237 | HEX (1) | 493 | 45 | 11.4 | 0.526 | 0.871 | 0.405 | 0.040 |
| 3 | N2724893 | (AAC)_10_ | 111–137 | ROX (1) | 539 | 10 | 5.5 | 0.505 | 0.635 | 0.212 | 0.076 |
| 4 | N2717495 | (TCTA)_10_ | 144–232 | HEX (2) | 510 | 20 | 8.8 | 0.405 | 0.840 | 0.507 | 0.058 |
| 5 | N178451 | (ATATA)_13_ | 97–188 | ROX (2) | 523 | 19 | 10.4 | 0.583 | 0.847 | 0.312 | 0.033 |
| 6 | N161850 | (AAT)_13_ | 102–192 | 6-FAM (3) | 531 | 31 | 15.3 | 0.716 | 0.872 | 0.167 | 0.041 |
| 7 | N49251 | (TCT)_11_ | 103–139 | HEX (3) | 543 | 13 | 7.0 | 0.637 | 0.742 | 0.150 | 0.076 |
| 8 | N350553 | (TTC)_22_ | 157–286 | HEX (3) | 505 | 43 | 11.6 | 0.460 | 0.734 | 0.399 | 0.105 |
| 9 | N935993 | (ATG)_10_ | 90–120 | ROX (3) | 512 | 12 | 6.5 | 0.626 | 0.686 | 0.093 | 0.162 |
| 10 | N2528349 | (ATCT)_20_ | 134–252 | HEX (4) | 543 | 31 | 8.0 | 0.461 | 0.612 | 0.235 | 0.119 |
| 11 | N2697375 | (TATG)_15_ | 136–220 | ROX (4) | 535 | 34 | 13.2 | 0.691 | 0.888 | 0.225 | 0.050 |

**Supplementary Table 7.** Estimates of multi-locus genetic diversity based on nuclear microsatellite dataset for 22 populations sampled. *N*_1_, number of 1-leaflet individuals genotyped; *N*_2_, number of 1-3 leaflets individuals genotyped; *N*_3_, number of 3-leaflets individuals genotyped; *N*_4_, number of 3-5 leaflets individuals genotyped; *N*_5_, number of 5-13 leaflets individuals genotyped; *N*, total number of individuals genotyped. *N*_a_, number of alleles; *H*_o_, observed heterozygosity; *H*_e_, unbiased expected heterozygosity; *F*_IS_, fixation index. For those genetic diversity parameters, data are presented as mean value averaged across 11 loci.

| **Population** | ***N*_1_** | ***N*_2_** | ***N*_3_** | ***N*_4_** | ***N*_5_** | ***N*** | ***N*_a_** | ***H*_o_** | ***H*_e_** | ***F*_IS_** |
| --- | --- | --- | --- | --- | --- | --- | --- | --- | --- | --- |
| **(1) *O. neimonggolica*** | **128** | **0** | **0** | **0** | **0** | **128** | **20.8** | **0.535** | **0.799** | **0.293** |
| N* | 34 | 0 | 0 | 0 | 0 | 34 | 13.1 | 0.531 | 0.771 | 0.272 |
| PFIF* | 29 | 0 | 0 | 0 | 0 | 29 | 9.9 | 0.506 | 0.666 | 0.239 |
| PSIT* | 34 | 0 | 0 | 0 | 0 | 34 | 12.5 | 0.528 | 0.769 | 0.286 |
| Y | 20 | 0 | 0 | 0 | 0 | 20 | 10.3 | 0.564 | 0.806 | 0.287 |
| Z | 11 | 0 | 0 | 0 | 0 | 11 | 7.8 | 0.552 | 0.747 | 0.273 |
| **(2) *O. diversifolia*** | **116** | **98** | **98** | **2** | **0** | **321** | **22.7** | **0.583** | **0.845** | **0.307** |
| PSET* | na | na | na | 0 | 0 | 7 | 5.4 | 0.572 | 0.772 | 0.273 |
| DIANO | 3 | 6 | 22 | 1 | 0 | 32 | 11.5 | 0.575 | 0.805 | 0.286 |
| HU | 4 | 21 | 7 | 0 | 0 | 32 | 10.9 | 0.585 | 0.818 | 0.290 |
| PTWE | 4 | 11 | 17 | 0 | 0 | 32 | 12.5 | 0.585 | 0.824 | 0.290 |
| PELE | 14 | 3 | 13 | 0 | 0 | 30 | 11.2 | 0.555 | 0.824 | 0.327 |
| PSIX | 4 | 14 | 14 | 0 | 0 | 32 | 12.5 | 0.614 | 0.841 | 0.273 |
| DIANT | 10 | 13 | 10 | 0 | 0 | 33 | 12.4 | 0.599 | 0.836 | 0.282 |
| PSEV | 11 | 12 | 9 | 0 | 0 | 32 | 12.1 | 0.644 | 0.840 | 0.233 |
| PE | 13 | 9 | 6 | 1 | 0 | 29 | 12.9 | 0.561 | 0.843 | 0.336 |
| GXB | 18 | 2 | 0 | 0 | 0 | 20 | 10.4 | 0.579 | 0.827 | 0.301 |
| DT | 17 | 5 | 0 | 0 | 0 | 22 | 10.3 | 0.562 | 0.831 | 0.312 |
| BT | 18 | 2 | 0 | 0 | 0 | 20 | 7.4 | 0.509 | 0.795 | 0.350 |
| **(3) *O. leptophylla*** | **0** | **1** | **0** | **5** | **96** | **102** | **13.1** | **0.495** | **0.729** | **0.319** |
| BOPO | 0 | 1 | 0 | 1 | 18 | 20 | 7.0 | 0.460 | 0.669 | 0.308 |
| BOPT | 0 | 0 | 0 | 1 | 18 | 19 | 8.0 | 0.548 | 0.715 | 0.241 |
| ZHONG | 0 | 0 | 0 | 0 | 22 | 22 | 7.9 | 0.487 | 0.729 | 0.333 |
| H | 0 | 0 | 0 | 2 | 19 | 21 | 6.6 | 0.461 | 0.697 | 0.344 |
| L | 0 | 0 | 0 | 1 | 19 | 20 | 7.1 | 0.517 | 0.730 | 0.295 |
| **Total** | **244** | **99** | **98** | **7** | **96** | **551** | **18.9** | **0.538** | **0.791** | **0.339** |

* An asterisk indicates a population with only leaves collected for phenotyping and genotyping. No information of microhabitat is available.

**Supplementary Table 8.** Inter- and intraspecific summary statistics for microhabitat variables estimated from 18 *Oxytropis* populations. Data are presented as mean (s.d.). *N*, number of individuals. *P*-values indicate the significance of differences among species (interspecies) or among phenotypes (intraspecies) in linear mixed-effects models, and percentage variance explained by the random factor “population” is given. Different letters indicate significant differences of pairwise comparisons (*P* < 0.05, Tukey’s HSD test).

|  | **Interspecies** | | | | |  | **Intraspecies (*O. diversifolia*)** | | | | |
| --- | --- | --- | --- | --- | --- | --- | --- | --- | --- | --- | --- |
|  | ***O. neimonggolica*** | ***O.***  ***diversifolia*** | ***O.***  ***leptophylla*** | ***P*-value** | **Pop.** |  | **1 leaflet** | **1-3 leaflets** | **3 leaflets** | ***P*-value** | **Pop.** |
|  | ***N* = 31**  **(1 leaflet)** | ***N* = 312**  **(1,1-3,3leaflets)** | ***N* = 95**  **(5-13leaflets)** |  |  |  | ***N* = 116** | ***N* = 98** | ***N* = 98** |  |  |
| **Slope (°)** | **14.9 (11.2) a** | **4.94 (3.64) b** | **13.1 (9.94) a** | **0.0055** | **44.8%** |  | **6.28 (4.77) a** | **3.89 (2.27) b** | **4.41 (2.58) b** | **0.076** | **36.0%** |
| Rocky ground (%) | 43.0 (16.6) | 22.2 (18.9) | 26.3 (16.6) | 0.16 | 39.8% |  | 25.5 (20.5) | 17.3 (16.2) | 23.2 (18.6) | 0.40 | 36.7% |
| Vegetation cover (%) | 27.1 (11.7) | 28.8 (18.8) | 33.3 (15.2) | 0.82 | 50.0% |  | 34.0 (21.7) | 27.9 (16.4) | 23.5 (15.5) | 0.71 | 54.8% |
| Bare ground (%) | 29.9 (16.5) | 49.0 (21.9) | 40.3 (16.3) | 0.44 | 56.6% |  | 40.5 (25.5) | 54.7 (18.9) | 53.3 (16.4) | 0.19 | 55.9% |

**Supplementary Table 9.** Summary of the likelihood-ratio test performed for each explanatory variable to explain leaflet-blade size indicators (individual-based model, *N* = 231). GLM have been performed using the *spaMM* package. All explanatory variables are covariates and *df* = 1.

| Variable | Early leaf length | | Mature leaf length | | Early leaf width | | Mature leaf width | |
| --- | --- | --- | --- | --- | --- | --- | --- | --- |
|  | Chi-square | *P*-value | Chi-square | *P*-value | Chi-square | *P*-value | Chi-square | *P*-value |
| Annual mean temperature (Bio01) | 0.53 | 0.46 | 1.83 | 0.18 | 0.76 | 0.38 | 0.023 | 0.88 |
| Mean diurnal range (Bio02) | **7.80** | **<0.01** | **6.73** | **<0.01** | 2.86 | 0.09 | 0.049 | 0.83 |
| Isothermality (Bio03) | **15.72** | **<0.0001** | **14.23** | **<0.001** | 2.78 | 0.10 | 1.97 | 0.16 |
| **Temperature seasonality (Bio04)** | **20.42** | **<0.0001** | **16.65** | **<0.0001** | **7.81** | **<0.01** | **6.91** | **<0.01** |
| **Annual precipitation (Bio12)** | **19.61** | **<0.0001** | **11.67** | **<0.001** | **11.60** | **<0.001** | **18.10** | **<0.0001** |
| Precipitation seasonality (Bio15) | 0.19 | 0.67 | **10.39** | **<0.01** | 0.00092 | 0.98 | 0.094 | 0.76 |
| STRUCTURE admixture proportion (*K* = 4) | 2.26 | 0.13 | **5.31** | **<0.05** | **4.87** | **<0.05** | 0.26 | 0.60 |
| Altitude | 0.055 | 0.82 | 0.0024 | 0.96 | 0.022 | 0.88 | 0.0014 | 0.97 |

**Supplementary Table 10.** Summary of the likelihood-ratio test performed for each explanatory variable to explain leaflet-blade size indicators (individual-based model, *N* = 231), **using aridity index instead of annual precipitation**. GLM have been performed using the *spaMM* package. All explanatory variables are covariates and *df* = 1.

| Variable | Early leaf length | | Mature leaf length | | Early leaf width | | Mature leaf width | |
| --- | --- | --- | --- | --- | --- | --- | --- | --- |
|  | Chi-square | *P*-value | Chi-square | *P*-value | Chi-square | *P*-value | Chi-square | *P*-value |
| Annual mean temperature (Bio01) | 1.01 | 0.31 | 1.27 | 0.26 | 1.19 | 0.27 | 0.022 | 0.88 |
| Mean diurnal range (Bio02) | **7.15** | **<0.01** | **6.51** | **<0.05** | 2.56 | 0.11 | 0.014 | 0.91 |
| Isothermality (Bio03) | **14.66** | **<0.001** | **13.79** | **<0.001** | 2.28 | 0.13 | 1.38 | 0.24 |
| **Temperature seasonality (Bio04)** | **19.10** | **<0.0001** | **15.96** | **<0.0001** | **6.87** | **<0.01** | **5.40** | **<0.05** |
| **Aridity index** | **19.51** | **<0.0001** | **12.03** | **<0.001** | **11.64** | **<0.001** | **17.77** | **<0.0001** |
| Precipitation seasonality (Bio15) | 0.45 | 0.50 | **11.22** | **<0.001** | 0.043 | 0.84 | 0.0032 | 0.95 |
| STRUCTURE admixture proportion (*K* = 4) | 2.31 | 0.13 | **5.44** | **<0.05** | **4.89** | **<0.05** | 0.25 | 0.62 |
| Altitude | 0.14 | 0.71 | 0.032 | 0.86 | 0.0010 | 0.97 | 0.012 | 0.91 |

**Supplementary Table 11.** Results of macroclimatic-association analysis for *O. diversifolia* based on stepwise multiple linear regressions, **using aridity index instead of annual precipitation**. Standardized coefficients estimated for the macroclimatic variables are shown. PV1, percentage of variation explained by temperature seasonality; PV2, percentage of variation explained by aridity index; PV3, percentage of variation explained by isothermality. ns, not significant, *P* > 0.10; + *P* > 0.05; * *P* < 0.05; ** *P* < 0.01; *** *P*< 0.001.

|  | Temperature seasonality | PV1  (%) | Aridity Index | PV2  (%) | Isothermality^a^ | PV3  (%) | Multiple *R*^2^ | *F*-statistic | *P*-value |
| --- | --- | --- | --- | --- | --- | --- | --- | --- | --- |
| (A) Population-based model (*N* = 12) | | | | | | | | | |
| Early leaf length | **-1.007***** | **42.3** | 1.094*** | 9.6 | **-0.936***** | **41.3** | 0.932 | *F*_3,8_ = 36.81 | <0.0001 |
| Mature leaf length | **-0.900**** | **35.3** | 0.973** | 4.8 | **-0.913**** | **39.2** | 0.794 | *F*_3,8_ = 10.25 | <0.01 |
| Early leaf width | -0.661** | 4.2 | **1.176***** | **48.4** | 0.656** | 29.8 | 0.824 | *F*_3,8_ = 12.50 | <0.01 |
| Mature leaf width | -0.992*** | 24.5 | **1.338***** | **47.6** | -0.711*** | 23.8 | 0.959 | *F*_3,8_ = 61.97 | <0.0001 |
| (B) Individual-based model (*N* = 213) | | | | | | | | | |
| Early leaf length | **-1.017***** | **16.2** | 0.712*** | 7.5 | **-1.242***** | **20.3** | 0.450 | *F*_4,196_ = 40.10 | <0.0001 |
| Mature leaf length | **-1.349***** | **11.2** | 0.558*** | 7.2 | **-1.869***** | **17.6** | 0.431 | *F*_6,197_ = 24.82 | <0.0001 |
| Early leaf width | -0.416*** | 6.1 | **0.539***** | **23.6** | ns | ns | 0.304 | *F*_3,197_ = 28.61 | <0.0001 |
| Mature leaf width | -0.409*** | 5.8 | **0.492***** | **22.2** | ns | ns | 0.280 | *F*_2,201_ = 39.11 | <0.0001 |

^a^ For early leaf width, the macroclimatic variable is annual mean temperature.
